# Selection of short Gadd45β‐binding peptides through a synergistic computational and biophysical approach

**DOI:** 10.1002/pro.70380

**Published:** 2025-11-18

**Authors:** Samuele Di Cristofano, Emanuela Iaccarino, Andrea Caporale, Daniela Verzella, Lucia Falcigno, Gabriella D'Auria, Rosita Russo, Camilla Rega, Angela Chambery, Angela Oliver, Giovannina Barisciano, Simon Cross, Gabriele Cruciani, Daria Capece, Francesca Zazzeroni, Menotti Ruvo, Annamaria Sandomenico, Domenico Raimondo

**Affiliations:** ^1^ Department of Molecular Medicine Laboratory Affiliated to Istituto Pasteur Italia‐Fondazione Cenci Bolognetti, Sapienza University of Rome Rome Italy; ^2^ Institute of Biostructures and Bioimaging (IBB) National Research Council (CNR) Naples Italy; ^3^ Institute of Crystallography (IC) National Research Council (CNR) Trieste Italy; ^4^ Department of Biotechnological and Applied Clinical Sciences University of L'Aquila L'Aquila Italy; ^5^ Department of Pharmacy University Federico II of Naples Naples Italy; ^6^ Department of Environmental, Biological and Pharmaceutical Science and Technology University of Campania “Luigi Vanvitelli” Caserta Italy; ^7^ Molecular Discovery, Kinetic Business Centre Borehamwood UK; ^8^ Department of Chemistry, Biology and Biotechnology University of Perugia Perugia Italy

**Keywords:** binding affinity, computational modeling, D‐tripeptides, Gadd45β, molecular dynamics simulations, protein–peptide interaction, saturation transfer difference nuclear magnetic resonance (STD‐NMR), therapeutic target

## Abstract

In this study, we explored the design of linear D‐tripeptides tailored to bind specific cavities of Gadd45β, chosen as a model protein target. To identify peptides that selectively interact with predicted binding sites, we combined computational modeling with biophysical experiments. Gadd45β was selected since it has emerged as a promising therapeutic target involved in multiple disease pathways, including cancer and inflammation. Computational analysis was first employed to characterize the structural features and potential binding sites of Gadd45β. Guided by these insights, linear D‐tripeptides were designed and optimized for specific interactions with the target surface. The resulting candidates were subsequently assessed through a series of biophysical assays to evaluate their binding affinity, selectivity, and potential therapeutic activity. Complementary computational simulations were employed to gain atomistic insight into the dynamics of peptide–protein recognition. This integrated computational–experimental strategy led to the identification of two D‐tripeptides, RYR and VWR, that bind Gadd45β at a biologically relevant site, illustrating a general framework for early‐stage peptide ligand discovery.

## 1 INTRODUCTION

Peptides have emerged as a suggestive frontier in drug discovery, offering the possibility to explore a unique chemical space between small molecules and biologics, with the potential to harness the advantages of both (Kaspar and Reichert 2013; Muttenthaler et al. 2021; Nielsen et al. 2017). Advances in parallel and combinatorial peptide synthesis, in structural modification, and in machine learning‐based predictions have accelerated peptide drug discovery (Romagnoli et al. 2025). However, traditional combinatorial chemistry approaches for ligand identification often face limitations, including the cost of synthesizing large numbers of single molecules and the risk of producing side products in mixture‐based screenings.

Despite the advent of artificial intelligence and machine learning approaches which have made available enormous computing power and increasingly sophisticated algorithms for predicting molecular interactions and thus filtering and reducing the number of potential hits, the process remains complex and still dependent on the need to perform wet screenings. On the other hand, massive wet random screenings, still used by pharma companies, require large economic resources for both acquisition and maintenance of compound libraries and for in vitro screening (Warr et al. 2022). Alternative approaches, based on screening combinatorial mixtures of synthetic or phage surface‐expressed peptides, while requiring less experimental effort and fewer resources in the early stages of the process, have often resulted in molecules quite large and difficult to depeptidize into bioactive compounds more similar to small molecules. As suggested elsewhere (Bedart et al. 2024), the most productive solution might be a balanced combination of in silico screening approaches, targeted use of AI, and simple and fast parallel synthesis methods, such as solid‐phase synthesis of small peptides, which is within the reach of any laboratory.

Short peptides offer numerous advantages over their larger analogues. Notably, they provide cost‐effective synthesis at both small and large scales, wide chemical diversity that can be expanded using non‐natural residues, the possibility of easy modification, high bioactivity, easy synthetic accessibility, and tunable functionalization. Due to their safe metabolites which are typically amino acids, and the limited potential for accumulation in the body, this type of molecule also features high biocompatibility, high safety, low toxicity as well as low immunogenicity (Perlikowska 2021; Soudy et al. 2022; Yang et al. 2023). Furthermore, they offer the additional advantage of immediate sequence optimization in a cost‐effective and timely manner.

With these key concepts in mind, we have developed a simplified approach to drug development that integrates in silico screening of small D‐tripeptides targeting protein cavities, chemical synthesis, and biophysical analyses based on robust techniques such as SPR and NMR. Small D‐tripeptides possess protease‐insensitive, small molecule‐like structures that, as such, can be seen as precursors of small molecule analogues with improved stability and reduced conformational flexibility.

Here, we present results from a case study where we have selected a ranked set of all‐D short peptide binders of Gadd45β (Zhan et al. 1994), addressing some of the limitations of traditional random screening methods.

The Gadd45 protein family, consisting of Gadd45α (Gadd45), Gadd45β (originally termed Myd118), and Gadd45γ (CR6) splicing isoforms, comprises small (~18 kDa), enriched in acidic residues (~16–17% of the total, resulting in a distinctly negative net charge at physiological pH) primarily localized within the cell nucleus (Zhan et al. 1994). These proteins play critical roles in stress signaling, influencing processes such as apoptosis, cell cycle arrest, DNA repair, cell survival, and senescence (Palomer et al. 2024; Salvador et al. 2013; Tamura et al. 2012; Wang et al. 1999). Gadd45 proteins share 55%–57% sequence identity at the amino acid level and lack enzymatic activity (Liebermann and Hoffman 2003; Papa et al. 2007).

Gadd45β regulates a broad array of biological processes through protein–protein interactions within both the nucleus and cytoplasm, highlighting its diverse roles in cellular physiology and pathology (Chathuranga et al. 2023; Liebermann and Hoffman 2008; Wu et al. 2024; Yang et al. 2009). Significantly, Gadd45β interacts with numerous proteins, including CDK1, Cyclin B1, p21, MTK1, MKK7, and Rb, affecting key pathways like JNK signaling and p38‐mediated activation (Bulavin et al. 2003; Jiang et al. 2022; Smith et al. 1994; Smith et al. 2000; Takekawa and Saito 1998; Vairapandi et al. 2000; Vairapandi et al. 2002). Gadd45β is a small (160 amino acids), multifunctional protein whose experimental 3D structure is still unknown. However, several predictions have suggested it to contain a four‐stranded beta‐sheet core, five alpha helices, and two acidic loops (aL1, residues 62–68; aL2, residues 103–117) (Papa et al. 2007; Tornatore et al. 2008; Tornatore et al. 2014a). These loops are critical for its function, particularly in inhibiting cdc223 and MKK7, with the A60‐D86 fragment playing a key role in binding and blocking MKK7 activity (Chathuranga et al. 2023; Papa et al. 2007; Rega et al. 2018). Additional key residues for MKK7 interaction are in helix 3 (H3), aL2, and part of helix 4 (H4) (Rega et al. 2018).

Due to its overexpression in tissues affected by various diseases, Gadd45β is considered a promising therapeutic target. The development of DTP3, a D‐tripeptide that binds MKK7 and inhibits the Gadd45β/MKK7 complex, underscores Gadd45β's therapeutic potential (Dubas n.d.) but also affirms the principle that protein cavities can be successfully targeted with small molecule‐like peptides that, taking advantage of the conformational flexibility of their backbone, explore at best their interaction capabilities. DTP3 is currently under clinical evaluation (The UK's Clinical Study Registry; https://doi.org/10.1186/ISRCTN13777452) as a drug to treat Multiple Myeloma (MM).

Since the biological functions of Gadd45β are only mediated by its protein interactions (Jiang et al. 2022), their modulation through small ligands is a priority in ongoing research, especially with a view to obtaining precursors for the design of small molecule drugs and selective probes for bioassays (Baroni et al. 2007; Stein and Kortemme 2013; Varadi et al. 2022).

## 2 RESULTS AND DISCUSSION

### 2.1 Gadd45β model building and conformational ensemble generation

Despite its biological relevance in physiology and pathology, experimentally determined Gadd45β structures are not yet available. To start investigating the binding modes of anti‐Gadd45β tripeptides, we first predicted the 3D structure of wild‐type Gadd45β using a local installation of AlphaFold2. To obtain the model, we thus input the primary sequence of the protein retrieved from the UniProt database (ID: O75293). Except for the N‐terminal tail (residues 1–15) and Acidic Loop 2 (aL2) (residues 103–117), the per‐residue confidence score (i.e., predicted local distance difference test, pLDDT) of the model was higher than 90 (pLDDT ≥90 indicates residues predicted with extremely high confidence) or between 70 and 90 (residues with 90 > pLDDT ≥ 70 are classified as confident). The N‐terminal tail and aL2 exhibited pLDDT scores lower than 70 (residues with 70 > pLDDT ≥ 50 are predicted with low confidence) (Jumper et al. 2021; Varadi et al. 2022) (Figure S1a, Supporting Information). To obtain a computationally tractable system and enable statistically meaningful sampling, we thus removed the N‐terminal tail of Gadd45β while aL2 was rebuilt de novo using Rosetta Next‐Generation KIC (Stein and Kortemme 2013) obtaining the model reported in Figure S1b.

To refine our Gadd45β model and sample the conformational ensemble of the protein, we employed all‐atom MD simulation in explicit TIP4P water using the Amber99SB‐ILDN force field (Figure S1c,e). A conformational ensemble consisting of 10 structures (shown in Figure S1d) was subsequently utilized for the ensemble docking approach.

In addition to AlphaFold2, we also explored both more traditional homology modeling approaches, such as MODELER, and more recent deep‐learning–based predictors, including AlphaFold3 and Boltz‐2.2 (Figure S2a,b).

To assess the consistency among these models, we performed a detailed comparative analysis by calculating the RMSD of the structured regions, explicitly excluding the flexible loops. As shown in the structural superposition (Figure S2b) and RMSD matrix (Figure S2c), all modeling methods yielded highly similar folds for the structured core of Gadd45β, with an RMSD always less than 0.2 Å. The largest deviations, as expected, were confined to the highly flexible acidic loop regions (Figure S2b).

### 2.2 Computational identification of Gadd45β cavities and D‐tripeptide design

**FIGURE 1:**
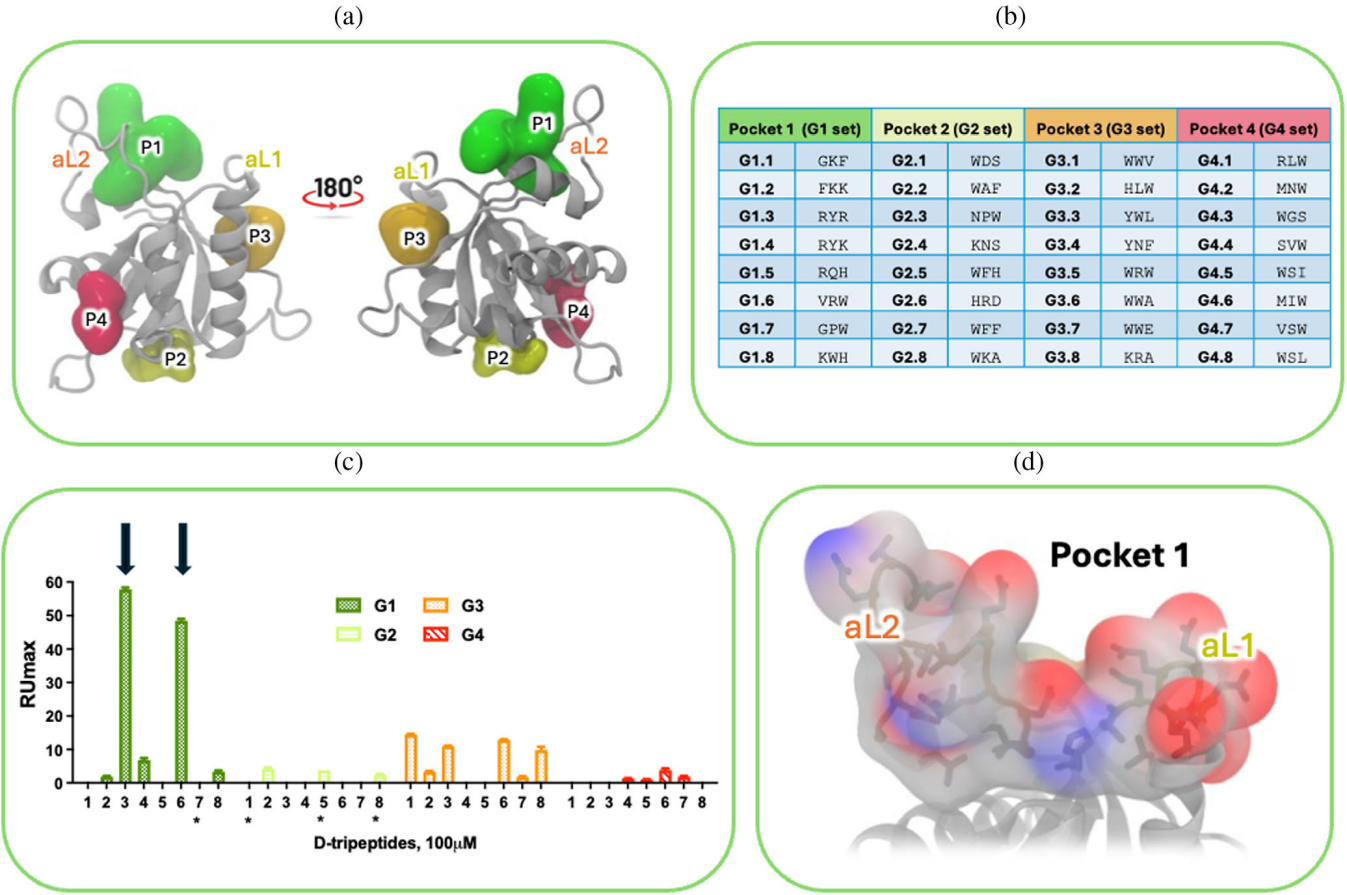
(a) Cavities identified on the predicted Gadd45β structure using FLAPsite, namely P1 (Pocket 1, green), P2 (Pocket 2, yellow), P3 (Pocket 3, orange), and P4 (Pocket 4, red). Acidic loops 1 and 2 are indicated as aL1 and aL2. (b) Table of sequences and codes of Gadd45β targeting D‐tripeptides designed using FLAPdock. All peptides were designed and prepared using the corresponding residues in the D‐configuration and as amidated C‐termini. (c) SPR label free binding data of the 32 designed D‐tripeptides. (d) Detail of P1 pocket. Gadd45β is depicted in cartoon, while the residues delineating P1 pocket are represented in sticks and surface. Red and blue colors indicate oxygen and nitrogen atoms, respectively.

In our effort to target the Gadd45β protein with D‐tripeptide active binders, we employed the FLAP*site* algorithm (integrated in FLAP) (Baroni et al. 2007; Siragusa et al. 2015) to explore all potential cavities in Gadd45β. This process generated accessible pockets as PDB files. Four distinct cavities, P1–P4, were detected (Figure 1a). The P1 cavity (represented in green in Figure 1a) was predicted between the two acidic loops, aL1 and aL2, while the P3 cavity is in proximity to aL1 (represented in orange in Figure 1a). These regions, described as the target sites for some therapeutically relevant interactors including MKK7, could suitably accommodate small‐molecules able to prevent their binding to Gadd45β (Rega et al. 2018).

To verify whether the identified binding pockets, particularly Pocket 1, are structurally or electrostatically unique to Gadd45β compared to other isoforms, despite Gadd45 proteins share more than 50% sequence similarity, we performed a detailed sequence‐ and structure‐based analysis of the three proteins of the Gadd45 family (Figure S3). Gadd45β possesses two acidic loops characterized by particularly pronounced biochemical properties: Residues 62–68 comprise a contiguous heptad of negatively charged side chains (DEEEEDD), while residues 103–117 (GEPAETQGTTEARDL) contribute an additional, more dispersed set of acidic residues (three Glu and one Asp). This primary‐sequence architecture creates a bipartite acidic surface that, in Gadd45β, is uniquely poised to present a concentrated electrostatic field within a confined volume between the two loops.

Multiple‐sequence alignment (MSA) (Figure S1) and 3D structure comparison (Figure S3b–d) reveal that this contiguous, high‐density acidic motif is a distinguishing feature of the Gadd45β protein (Figure S3a,b). In Gadd45α the analogous region contains acidic residues but they are interrupted by substitutions that reduce local charge continuity or introduce basic charges (e.g., R67) (Figure S3a,c); Gadd45γ commonly shows deletions or non‐conservative replacements that disrupt the contiguous acidic stretch (Figure S3a,d). Thus, whereas all family members share an overall acidic patch in the central region, Gadd45β alone bears a tightly clustered, uninterrupted acidic motif that will amplify and localize negative potential relative to *α* and *γ*.

Structural pocket detection with the CASTp algorithm supports the functional consequence of this sequence distinction: CASTp identifies a substantial binding pocket between the two acidic loops in Gadd45β (volume = 394.233 Å^3^), whereas no pocket is detected at the corresponding location in the Gadd45α structure (PDB ID: 2KG4, NMR model n.1). Gadd45γ (PDB ID: 3FFM) presents only a very small cavity at the equivalent site (volume = 10.864 Å^3^); this striking difference is plausibly a consequence of loop orientation and inter‐loop geometry in *γ*, where relative angulation or tighter apposition of the backbones collapses the inter‐loop cleft and prevents the formation of an accessible cavity. Collectively, the dense, contiguous acidic motif of Gadd45β, together with a well‐defined inter‐loop pocket of appreciable volume, provides a structurally and electrostatically distinctive binding site that rationalizes isoform‐specific recognition of basic ligands and highlights a clear target for selective inhibitor design.

To obtain binders for these pockets, we chose to start with D‐tripeptides, which, because of their size (all less than 500 Da) and the D‐configuration of their residues, are the ideal option for developing small‐molecule precursors. The D‐tripeptides targeting cavities P1–P4 were generated using a version of FLAP*dock* optimized for peptide docking. FLAP software was employed to generate starting poses for each amino acid. The tripeptide structure was expanded by sampling torsional space, and finally, the poses were minimized and scored within the cavities. FLAP*dock* was employed to dock the tripeptides, scoring the interactors by a combination of hydrophobic, electrostatic, H‐bond donor and acceptor molecular interaction fields (MIFs) similarity, along with classical energetic terms (further technical details are reported in section 4). The software produced four sets of D‐tripeptides ranked for their potential to complement the physico‐chemical properties of the target cavities. The screened peptides were 19^3^ = 6859, that is, all combinations of tripeptides with 19 of the 20 natural amino acids (excluding cysteine). The 25 best hits for each of the four cavities (25 tripeptides × 4 cavities = 100 D‐tripeptide sequences) were next selected for further investigation, though only the top 8 ranked peptides from each cavity were selected for synthesis and subsequent testing using label‐free techniques and biophysical methods (8 tripeptides × 4 cavities = 32 tripeptides). The tripeptide sets designed on the four Gadd45β cavities were named G1, G2, G3, and G4, respectively. Tripeptide sequences and targeted pockets for each set are listed in Figure 1b which for simplicity are reported as single upper‐case letters.

### 2.3 Testing the binding of designed tripeptides with Gadd45β

All 32 designed D‐tripeptides were prepared by chemical synthesis as C‐terminally amidated molecules and obtained in quantitative yield and with purities exceeding 95%, which is consistent with expectations for tripeptides. Peptides were purified by semi‐preparative RP‐HPLC as described elsewhere (Caporale et al. 2017) confirming identity and purity through LC–MS analyses.

**FIGURE 2:**
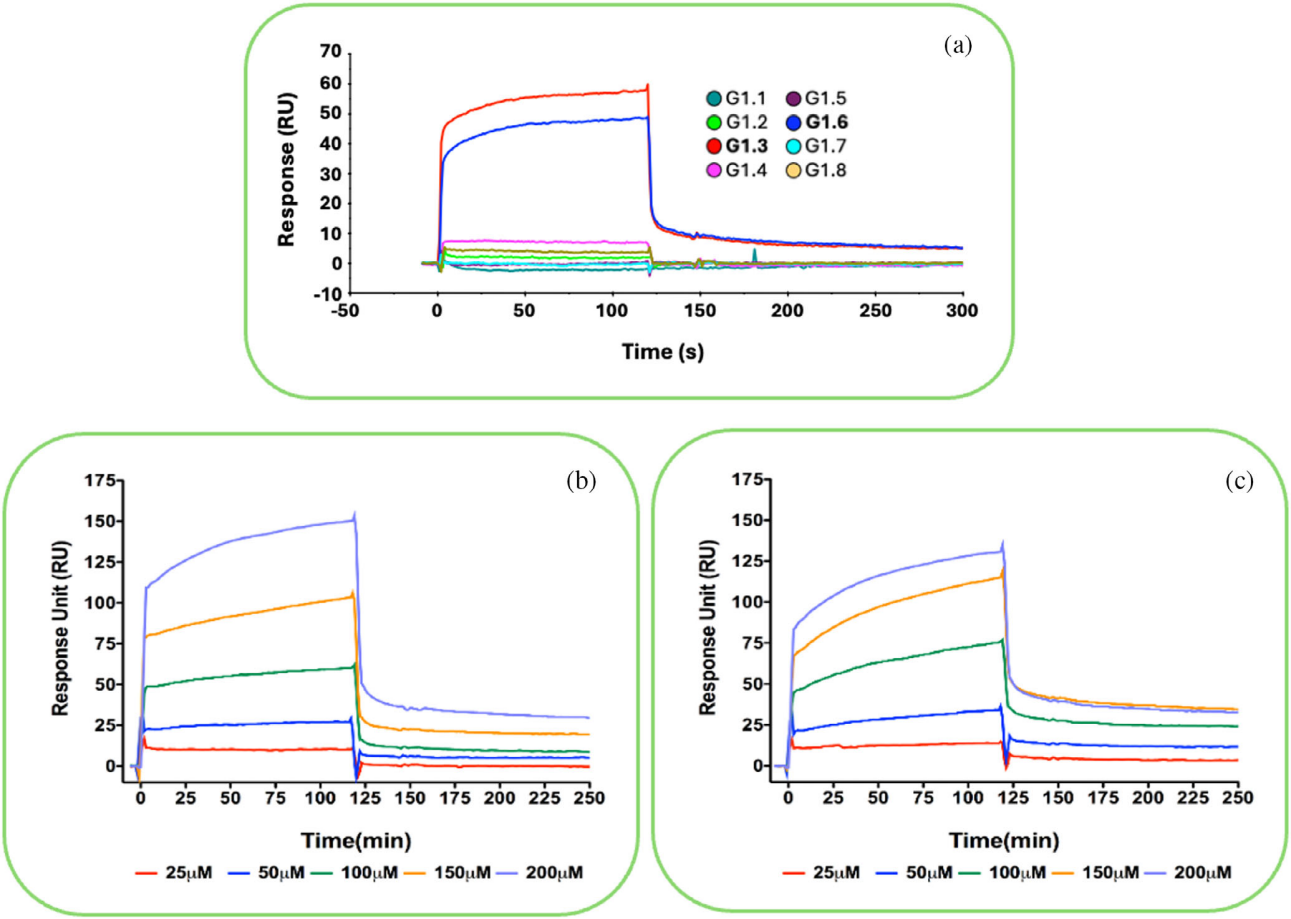
SPR screening assay. Screening of the 8 anti‐pocket1 D‐tripeptides for their ability to bind rhGadd45β immobilized on a CM5 sensor chip. Single D‐tripeptides were tested at 100 μM (a). Dose response assays of the two selected tripeptides G1‐3 (RYR) (b) and G1‐6 (VRW) (c) at concentrations ranging between 25 and 200 μM. SPR experiments were repeated twice, though only one of them is reported.

All peptides underwent biophysical investigations to identify those binding to Gadd45β. The initial screening was conducted through surface Plasmon resonance (SPR) using a Biacore 3000 instrument (Cytiva, Milano, Italy), testing their ability to bind rhGadd45β immobilized on the surface of CM5 sensorchips at the single concentration of 100 μM (Figure 2a). Data showed that molecules named G1.3 and G1.6, with sequences D‐Arg‐D‐Tyr‐D‐Arg‐NH_2_ and D‐Val‐D‐Arg‐D‐Trp‐NH_2_ (hereafter reported simply as RYR and VRW, in agreement with the notation of Figure 1b), respectively, resulted in the strongest binders for rhGadd45β (Figure 1c) in these conditions (Figure 2a). The G2 and G4 sets were essentially inactive, while some peptides of the G3 set exhibited weak binding signals. Given the major relevance of cavity P1 for the protein's biological activity, tripeptides targeting the other cavities were omitted in the subsequent biophysical investigations.

Following this first screening, we next tested the two best binders RYR and VRW D‐tripeptides in dose‐dependent assays to determine their affinity for the target protein and binding kinetics. As illustrated in Figure 2b,c (see also Tables 1 and 2), both RYR and VRW peptides exhibited dose‐dependent binding to the immobilized protein, demonstrating similar kinetics, with rapid association (*k*_on_ = 10^2^ M^−1^/s) and dissociation rates (*K*_off_ = 10^−3^ s^−1^). Through data fitting using BiaEvaluation software, dissociation constants (*K*_*D*_ *s*) of 2.75 ± 0.07 × 10^−5^ M for RYR (Table 1) and 2.19 ± 0.13 M × 10^−5^ (Table 2) for VRW was determined, respectively. The tripeptides FKK and RQH, also tested in this experiment, did not show any binding to the protein at the concentrations tested.

**TABLE 1:** Kinetic and affinity constants of H‐RYR binding to rhGadd45β.

	*K*_*a*_ (1/Ms)	*K*_*d*_ (1/s)	*K*_*D*_ (M)
25 μM	5.83*10^2^	4.83*10^−5^	2.18*10^−6^
50 μM	3.22*10^2^	4.39*10^−3^	1.36*10^−6^
100 μM	1.19*10^2^	2.45*10^−3^	2.06*10^−5^
150 μM	0.27*10^2^	2.29*10^−3^	8.38*10^−5^
200 μM	0.95*10^2^	2.43*10^−3^	2.55*10^−5^
Average μM	2.76*10^2^	2.07*10^−3^	2.75*10^−5^

**TABLE 2:** Kinetic and affinity constants of H‐VRW binding to rhGADD45β.

	*K*_*a*_ (1/Ms)	*K*_*d*_ (1/s)	*K*_*D*_ (M)
25 μM	2.06*10^2^	4.90*10^−3^	2.38*10^−5^
50 μM	1.43*10^2^	2.94*10^−3^	2.06*10^−5^
100 μM	0.93*10^2^	2.68*10^−3^	2.86*10^−5^
150 μM	0.86*10^2^	2.79*10^−3^	3.25*10^−5^
200 μM	0.87*10^2^	3.29*10^−3^	3.78*10^−6^
Average μM	1.23*10^2^	3.32*10^−3^	2.19*10^−5^

### 2.4 Structural characterization of Gadd45β engagement by the tripeptides

To complement the SPR label‐free screening and provide additional information about the peptide's structures contribution to Gadd45β binding, we undertook saturation transfer difference NMR (STD‐NMR) analysis on six out of the eight peptides included in the G1 set (G1.1 through G1.6; Figure 1b). The STD experiment is able not only to confirm the binding phenomenon but also to map the surface of the ligand that contacts the protein pocket. This experiment consists of performing two different 1D‐NMR spectra on a sample containing both protein (micromolar range) and ligand (in large excess) (Klein et al. 1999; Mayer and Meyer 1999; Mayer and Meyer 2001). In the first NMR experiment (called on‐resonance), the protein is selectively irradiated at a radio frequency close to the protein signals and far from any ligand signals. Prolonged irradiation causes the saturation of the proton resonances of the entire protein and of the bound ligands by spin diffusion. This on‐resonance spectrum contains only peptide signals whose intensities will be reduced depending on the saturation experienced. The second NMR experiment is designed to produce a protein plus peptide spectrum at full intensity (off‐resonance spectrum). The STD spectrum is then obtained by subtracting the two experiments from each other. The resulting difference spectrum shows only signals from protons bound to the protein. Moreover, the closer the protons are to the protein pocket, the stronger their signals will be in the STD spectrum.

**FIGURE 3:**
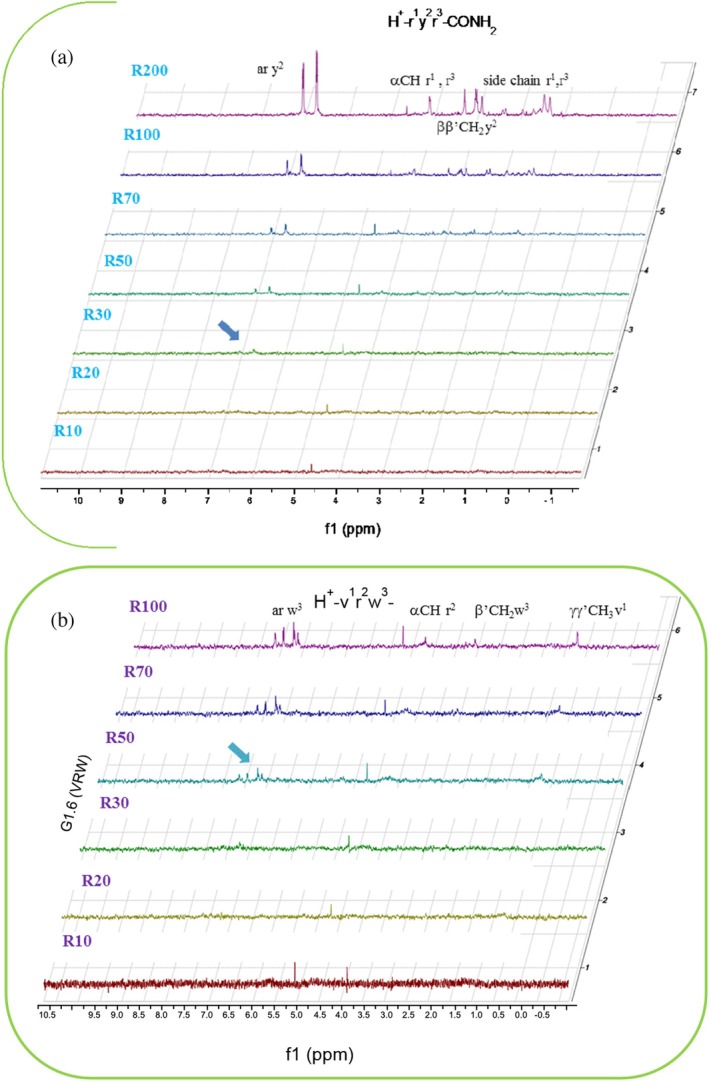
STD spectra of (a) G1.3 and (b) G1.6. G1 peptides/Gadd45β at increasing R molar ratios.

For each system peptide‐Gadd45β, a series of STD spectra was acquired by adding increasing quantities of peptide to the NMR tube containing a fixed amount of protein (20 μM), to reach peptide/protein molar ratios (R) ranging from 10 to 100 or 200. The comparison among the STD series of the six peptide‐protein systems allowed us to highlight the best binders. In Figures 3a,b and S13 the STD series of the six peptides are presented at increasing R ratios. As shown in Figure 3a,b, peptides RYR (G1.3) and VRW (G1.6) were confirmed as effective interactors of Gadd45β. However, peptides GKF (G1.1) and RYK (G1.4), showing poor interactions through SPR, also appear to interact with the protein (Figure S13a–d) at these high concentrations. Given the weak interaction signals shown by the SPR analyses they were not further investigated. Importantly, FKK (G1.2) and RQH (G1.5) are not binders for both techniques. The primary structures of binder and non‐binder peptides support the notion that the binding is not mediated by mere electrostatic interactions between the positively charged side chains of arginine and lysine with the extended negatively charged region of the protein. Rather, the STD peaks observed with the active peptides are strongly suggestive of interactions mediated by a combination of contacts involving aromatic and polar hot spots.

At higher ratios, some αCH protons and certain side‐chain protons also become evident in STD spectra. It is important to note that hydrogen atoms exhibiting higher intensities are those most directly involved in the binding process and are in closer proximity to Gadd45β. Insights on the ligand surface most involved in the Gadd45β contact were obtained through group epitope mapping (GEM) (Mayer and Meyer 2001). In the GEM analysis, STD integrals of individual protons are normalized to the most strongly interacting proton (integral value 100%), that is, the one at the shortest distance from the protein surface (Figure S4c). The GEM analysis confirms that the aromatic protons of Tyr, and Trp residues in RYR and VRW, respectively, are primarily involved in protein binding, followed by some αCH protons (Figure S4c). The STD‐NMR data prompted us to investigate the molecular determinants of the interaction between D‐tripeptides and Gadd45β through computational simulations of the two best binders, RYR and VRW, identified from both SPR and NMR analyses.

### 2.5 In silico analysis

A robust computational workflow was used to investigate the molecular mechanism and dynamics of interaction between Gadd45β and the two experimentally selected tripeptides. This involved ensemble docking simulations followed by extensive multiple independent unbiased molecular dynamics simulations at different temperatures, allowing us to identify a plausible final binding mode for each tripeptide (see scheme in Figure S6a–e).

By this analysis, both tripeptides RYR and VRW resulted in complementing the Gadd45β cavity designated as P1 (Figure 1a). This cavity is characterized by two parallel acidic loops that lack stable secondary structure elements and exhibit significant flexibility (Figure S1c–e). Also, the tripeptides exhibit high flexibility due to the presence of arginine residues, which harbor three CH_2_ groups in the side chain.

Ensemble docking approaches are particularly suited for binding processes involving significant conformational changes, as observed in our case, provided that all relevant protein conformations are considered. These methods involve ligand docking to predetermined multiple receptor conformations (MRC), which can be obtained from either experimental data (such as X‐ray crystallography or NMR spectroscopy) or computational sampling techniques (e.g., molecular dynamics, Monte Carlo simulations, or normal‐mode analysis). Here, we performed the so‐called relaxed complex scheme (RCS) in which the MRC are generated by MD simulations of the unbound Gadd45β protein (Figure S1c,e), followed by protein loop RMSD cluster analysis to select representative conformations (Figure S1d). The selection of reduced numbers of representative conformations from the MD trajectory is crucial for ensemble‐docking applications. Different variables can be considered to cluster distinct macrostates, but for the purpose of our ensemble docking simulation, a good clustering protocol should be able to recognize different arrangements of the cavity on which tripeptides are designed. Therefore, we employed two different clustering algorithms focused on the most flexible loop (aL2, residues 103–117) delineating the P1 cavity of Gadd45β: the hierarchical‐agglomerative clustering and *k*‐means clustering. Based on clustering quality metrics (see section 4) and visual inspection, we selected an ensemble consisting of 10 Gadd45β conformations, obtained from *k*‐means clustering, that better described the flexibility and sampled different degrees of opening/closing of the region of interest (Figure S1d).

Then, for each peptide (RYR and VRW) we conducted a series of independent docking runs to all members of the ensemble resulting in 90 binding poses for each D‐tripeptide. To streamline the amount of binding modes to a computationally manageable number, we filtered out all poses obtained from the ensemble docking simulations based on the STD NMR results. As reported above NMR experiments indicated that aromatic protons of RYR and VRW tripeptides are significantly involved in Gadd45β binding (Figure 3a,b). Therefore, according to this information we excluded poses that exhibited solvent‐accessible surface area (SASA) for tyrosine on RYR and tryptophan on VWR greater than 20 Å^2^. This exclusion criterion (defined as “*SASA filtering step*” in Figure S4a) was applied under the assumption that the interacting aromatic residues of the tripeptides should be buried within the pocket formed by the two acidic loops upon Gadd45β binding.

After the *SASA filtering step*, which retained 31 poses for RYR and 7 poses for the VRW peptide (Figure S4a), we selected the top‐ranking poses for each peptide using three distinct scoring functions (SFs) (Figure S4b): AutoDock Vina, CNNscore, and CNNaffinity. This process yielded three poses: pose #1, pose #2, and pose #3 for both RYR and VRW. Visual inspection of the RYR poses reveals that the positioning of the arginines toward αL1 is consistent across both binding modes (Figure S4b). In poses #1 and #3, the tyrosine side chain is relatively buried, but it is not stabilized by the helix α1 as observed in pose #2. The helix α3 in the corresponding Gadd45β conformation exhibits a greater degree of opening, allowing for an open conformation of RYR, positioning the arginine on the αL1 loop and the tyrosine within αL2. In the case of VRW, the arginine appears to establish interactions of varying strength with αL2, while the tryptophan is well buried and stabilized by αL2 only in pose #3.

### 2.6 Dynamic portrait of tripeptide/Gadd45β complexes revealed by extensive MD simulations

To gain further insights into the tripeptide's mechanism of interaction with the protein, we ran physiological and high‐temperature MD simulations starting from the top three scored complexes (Figure S4b); this experiment allowed us to comprehensively account for both solvent effects and reciprocal protein–peptide dynamics.

**FIGURE 4:**
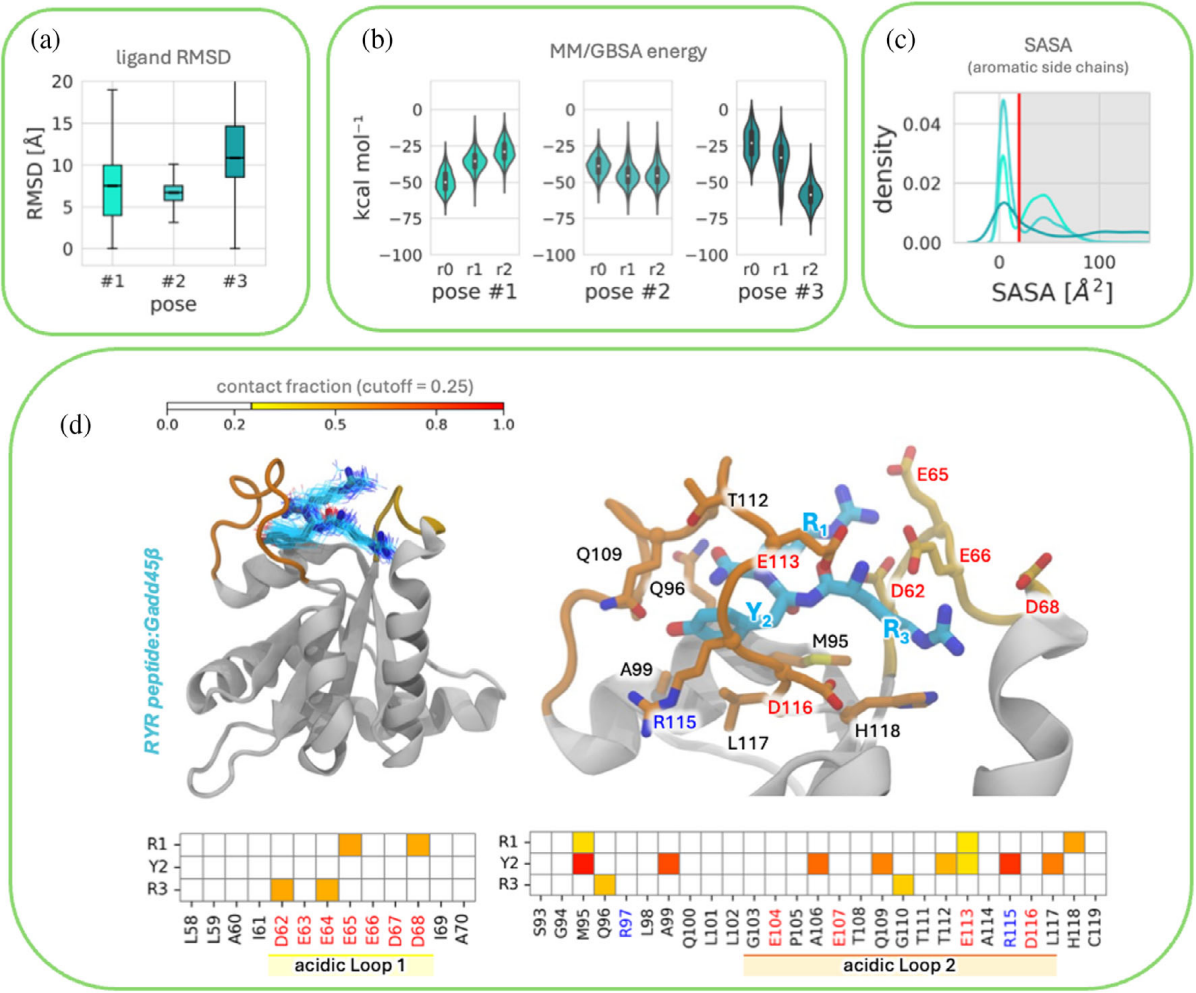
(a) Box plots of differences in ligand RMSD for three different RYR binding poses (pose #1, pose #2, and pose #3). The box indicates the middle two quartiles of the distribution (25 percentile to 75 percentile). The average of the distribution is shown by the line inside the box. Each pose was simulated in three MD replicas. (b) Violin plots illustrating the distribution of peptide:Gadd45β intermolecular binding energy across binding poses as measured using MM/GBSA analysis. Each pose was simulated in three MD replicas. (c) Distribution of observed SASA of Tyr residue in RYR during the simulations to assess the buriedness of the aromatic side chain. Each distribution results from the aggregation of the frames sampled across three replicates per each pose. Poses #1, #2, and #3 are represented by light cyan, cyan, and dark cyan distribution, respectively. (d) Frequency of RYR contacts as determined during MD simulations. 5000 frames representing the most stable binding mode (pose #2) were used for the analysis. Contacts less frequent than 25% of the analyzed frames were excluded from the analysis.

**FIGURE 5:**
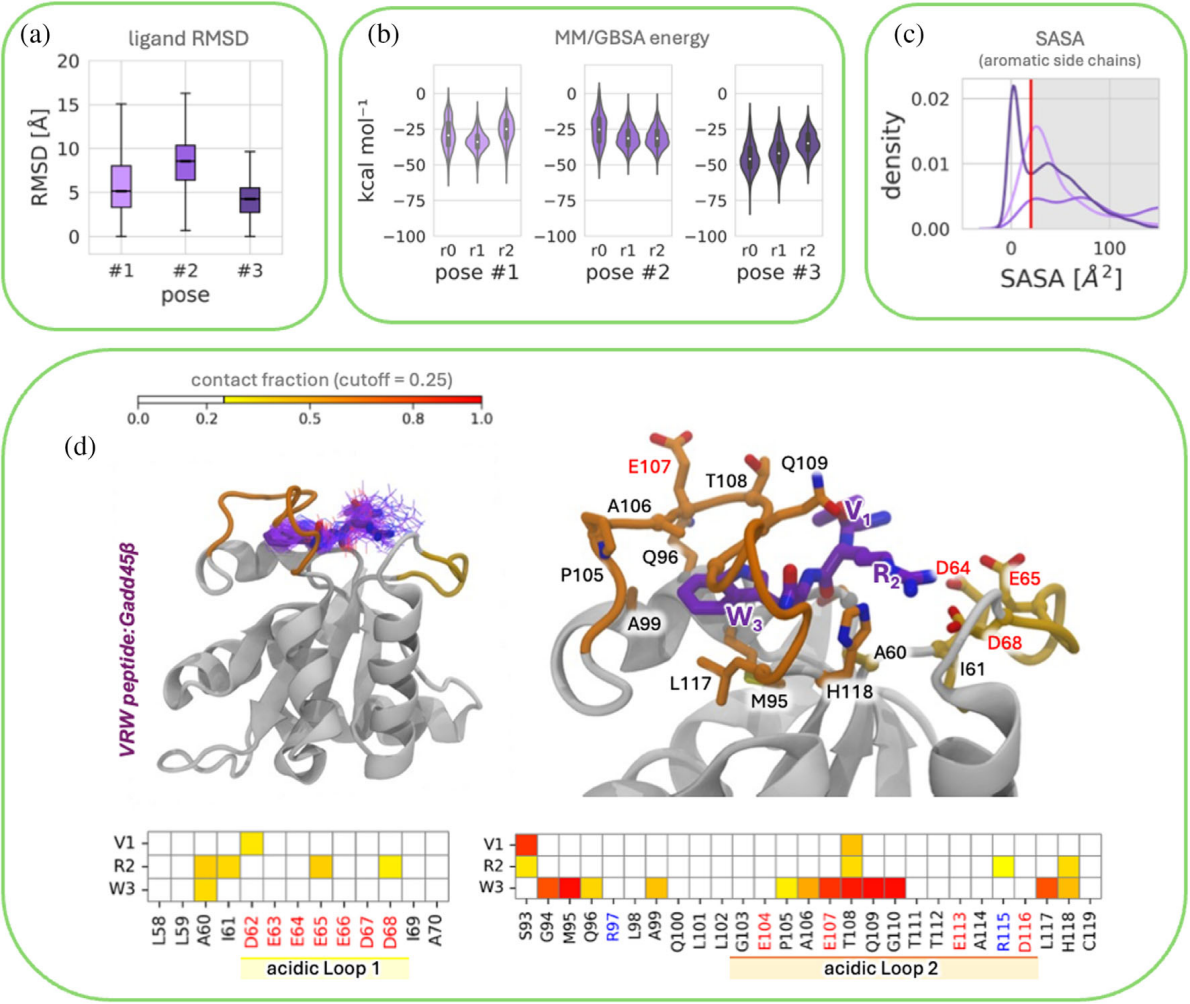
(a) Box plots of differences in ligand RMSD for three different VRW binding poses (pose #1, pose #2, and pose #3). The box indicates the middle two quartiles of the distribution (25 percentile to 75 percentile). The average of the distribution is shown by the line inside the box. Each pose was simulated in three MD replicas. (b) Violin plots illustrating the distribution of peptide:Gadd45β intermolecular binding energy across binding poses as measured using MM/GBSA analysis. Each pose was simulated in three MD replicas. (c) Distribution of observed SASA of Trp residue in RYR during the simulations to assess the buriedness of the aromatic side chain. Each distribution results from the aggregation of the frames sampled across three replicates per each pose. Poses #1, #2, and #3 are represented by light light purple, purple, and dark purple distribution, respectively. (d) Frequency of VRW contacts as determined during MD simulations. 5000 frames representing the most stable binding mode (pose #2) were used for the analysis. Contacts less frequent than 25% of the analyzed frames were excluded from the analysis.

Initially, the Gadd45β:tripeptide complexes underwent extensive MD simulations at physiological temperature (310 K). Specifically, a minimum of three independent replica MD simulations were conducted for pose #1, pose #2, and pose #3 for RYR and VRW respectively. This resulted in an aggregate sampling of over 5 μs at 310 K (2.7 μs for each binder). We utilized the ligand RMSD metric (ligand root‐mean‐square deviation from the starting pose) to assess the dynamic stability of different complexes (Figures 4a, 5a, and S5). This metric was calculated by aligning only the Cα atoms in secondary structures of the protein to the initial structure and then determining the ligand RMSD relative to its initial structure.

For both RYR and VRW tripeptides, ligand RMSD calculations indicated that the three poses showed different behaviors during MD simulations. For RYR, pose #2 exhibited the highest stability, as reflected by the lowest average and standard deviation of ligand RMSD values (mean ± SD = 6.3 ± 4 Å) (Figure 4a). For VRW, pose #3 exhibited the highest stability (ligand RMSD values, mean ± SD = 4.2 ± 1.7 Å) (Figure 5a).

Interestingly, examination of ligand RMSD traces over time (Figure S5a) revealed that in one of the three MD replicas (r3) of RYR pose #3, the RYR peptide moved far away from the protein at 50 ns, as evidenced by a sharp increase in ligand RMSD The peptide remained free in solution for approximately 50 ns before re‐binding to the Gadd45β P1 cavity at 100 ns inserting the tyrosine side chain into aL1 hydrophobic portion. Remarkably, the new binding mode closely resembled RYR pose #2 (Figure S5c) and remained stable for the remainder of the simulation. A similar behavior was also observed for RYR pose #3 replica 2 (r2) and pose #1 replica 1 (r1): in this case we notice that the transition toward pose #2 happened while the tripeptide remained in proximity to the acidic loops. This spontaneous transition from RYR pose #1 and pose #3 to RYR pose #2 may suggest the higher stability of RYR pose #2 compared to other poses.

Regarding VRW we did not observe any pose interconversion. However, it should be noted that these transitions were observed within replicate trajectories of approximately 900 ns for each peptide studied at physiological temperature, a timescale that may not fully capture slower or rarer binding events occurring over multiple microseconds. The observed transitions should thus be interpreted as indicative of local rearrangements within the accessible timescale.

Despite the apparent promise of RYR pose #2 and VRW pose #3 as the most stable candidates, none of the three binding modes of each peptide was excluded from further rescoring. In fact, we proceeded to calculate the MM/GBSA relative binding free energy to obtain an estimation of the interaction strength in all three poses (Figures 4b and 5b). The use of MM‐GBSA and/or MM‐PBSA and re‐ranking docking results is a well‐established practice, as these approaches often provide superior discriminative power compared to docking scores alone (De Vivo et al. 2016; Garibsingh et al. 2021).

We also monitored the solvent‐accessible surface area (SASA) of aromatic amino acid residues (Figures 4c and 5c). This served two purposes: (i) to validate the consistency between STD NMR experiments and our MD simulations, and (ii) to confirm the rationale behind the SASA filtering step previously applied to docking results. Indeed, subsequent equilibrium MM/GBSA protein–tripeptide binding affinity calculations were employed for ranking the stable poses. RYR pose #2 showed the highest binding affinity (mean ± SEM = − 43.3 ± 0.13 kcal/mol) followed by pose #3 (mean ± SEM = − 38.8 ± 0.21 kcal/mol) and pose #1 (mean ± SEM = − 37.9 ± 0.13 kcal/mol).

Therefore, according to our results, more stable binding poses with lower ligand RMSD consistently have better binding affinity values than less stable poses with higher ligand RMSD. It is worthwhile to note that the interconversion from replicas of RYR pose #1 (r1) and pose #3 (r2 and r3) to RYR pose #2 (Figure S5c) reflects the higher binding affinity reported in Figure 4b.

For these complexes MM/GBSA binding energy estimates completely confirm pose #3 as the strongest interacting pose (mean ± SEM = −40.3 ± 0.12 kcal/mol). Pose #1 and #2 exhibited MM/GBSA binding energy expressed as mean ± SEM equal to – 29.3 ± 0.11 kcal/mol and –27.3 ± 0.11 kcal/mol, respectively (Figure 5b). These estimates should be interpreted in a relative sense, since MM/GBSA binding free energies are not directly comparable with experimental values, as they do not account for all entropic and solvent contributions. Therefore, the results presented here provide a comparative internal ranking of poses rather than absolute affinity predictions.

SASA analysis of tyrosine on RYR and of tryptophan on VRW, computed along MD trajectories of all 3 binding poses (for each of the two peptides), demonstrated that the aromatic residues of their side chains are more buried in the poses resulting in more stability and show higher affinity to Gadd45β (i.e., RYR pose #2 and VRW pose #3) (Figure 4c).

This straightforward analysis of aromatic residue buriedness enabled us to validate the consistency between STD‐NMR and MD results and further confirmed the rationale behind the SASA filter we applied to docking results.

Finally, inter‐residue contact analysis was conducted to elucidate the molecular determinants of different binding affinities. In Figures 4d and 5d we report the results of the most stable poses for both D‐tripeptides (RYR pose #2 and VRW pose #3), while a complete overview of contact analysis for all the poses is reported in Figure S7.

The arginine residues in RYR pose #2 are stabilized by several hydrogen bonds/salt bridges with the protein acidic loops. In particular, the N‐terminal arginine of RYR interacts with residues Glu65 and Asp68 of protein αL1, while the one at the C‐terminus interacts with Asp62 and Glu64. Coherently with STD‐NMR data, the buried side chain of tyrosine of RYR is packed with Met95, Ala99, Ala106, and Leu117 establishing strong hydrophobic interactions, while its ‐OH group forms a hydrogen bond with Arg115 of αL2 (Figure 4d).

As previously described, the acidic properties of Gadd45β are conferred by two acidic loops spanning residues 62–68 (άL1) and 103–117 (αL2), respectively, which are essential for several interactions and functions. These residues collectively form a negatively charged “crown” that tightly binds the arginine residues of RYR, thereby restricting its conformational space (Figure 4). We support this hypothesis by comparing RMSF, in particular of the acidic loop, between the simulations with Gadd45β in isolation or in complex with the peptides (Figure S8). The results indicate that both RYR and VRW reduce the flexibility of the two loops. The effect on the smaller and less flexible αL1 is negligible, whereas αL2 is strongly stabilized upon peptide binding. These findings support our hypothesis regarding the role of peptide interactions in modulating loop dynamics.

Reflexively, the tight binding of RYR's arginine's side chains reduces the flexibility of the Acidic Loop. On the other hand, the N‐terminal valine residue of VRW in pose #3 is stabilized by Asp62 from αL1 and Ser93 and Thr108 from αL2. The arginine of VRW is mainly engaged through hydrogen bonds/salt bridges with Glu65 and Asp68 of αL1 and through hydrophobic interactions with Ala60 and Ile61. Similarly to RYR—and in agreement with NMR data—the VRW tryptophan is packed with Ala60, Gly94, Met95, Gln96, Ala99, Pro105, Ala106, Glu107, Thr108, Gln109, Gly110, Leu117, and His118 of the protein (Figure 5d). Finally, we further evaluated protein/peptide complexes besides the ligand RMSD, analyzing the interface RMSD (I‐RMSD), as used in the CAPRI competition (Lensink and Wodak 2013). The corresponding results are presented in Figure S9. Notably, the same trend observed in the ligand RMSD, MM/GBSA re‐scoring, and aromatic side chain SASA analyses (Figures 4a–c and 5a–c) is also reproduced.

### 2.7 Capturing thermal stability of Gadd45β/tripeptide complexes through high‐temperature MD simulations

Successful strategies for characterizing protein–peptide systems require the ability to account for flexibility to sample bound conformations and accurate functions that distinguish the best binders among a series of peptide candidates.

**FIGURE 6:**
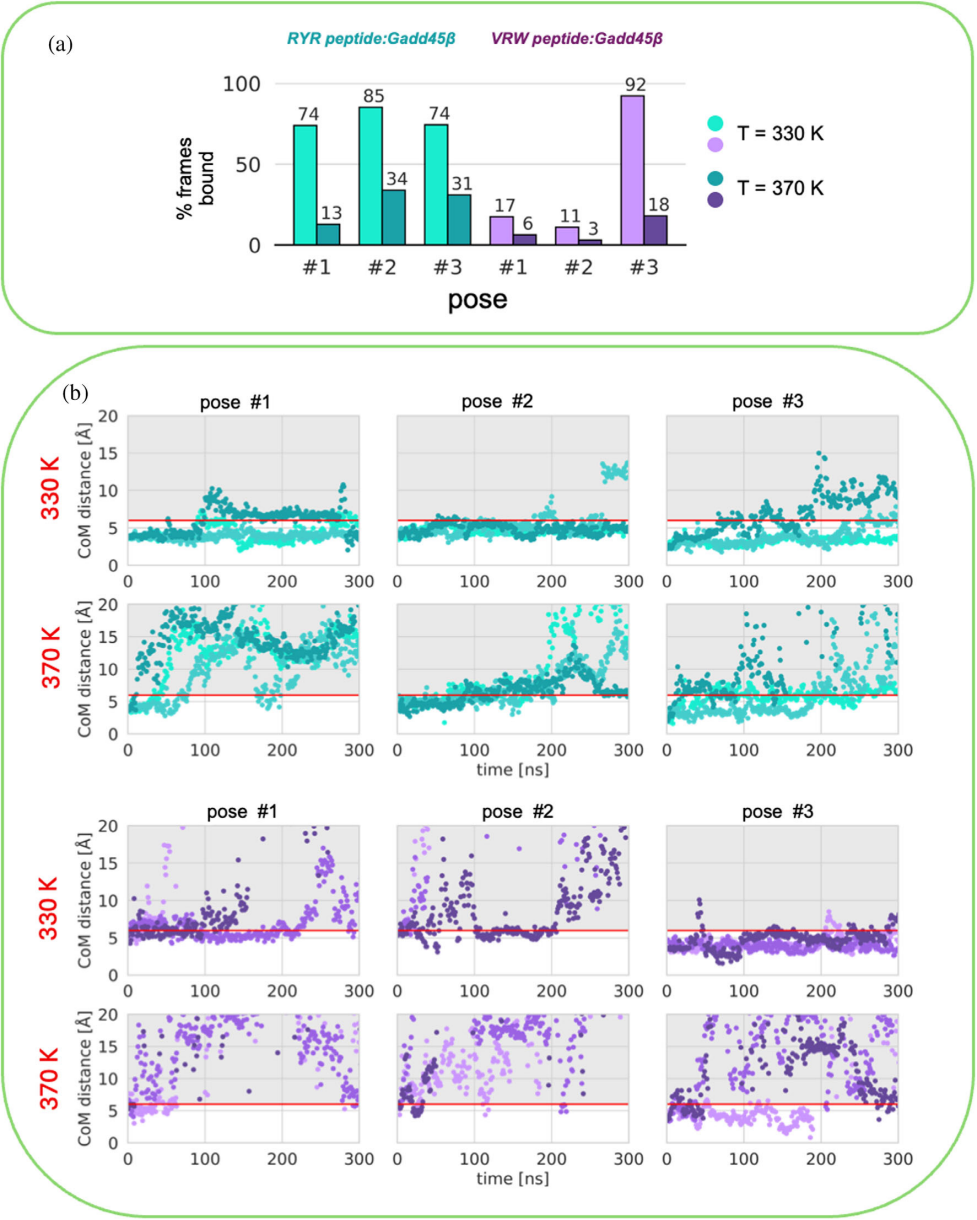
(a) Bar plot showing the percentage of bound frames for the selected three poses of RYR and VRW at 330 and 370 K. Light colors indicate simulations conducted at 330 K; dark colors indicate 370 K MD simulations. (b) Center of mass (CoM) distance as a function of simulation time computed between peptide and pocket Cα atoms. Horizontal red line corresponds to the 6 Å COM distance cutoff used to define bound/unbound frames. Poses #1, #2, and #3 are represented in light cyan, cyan, and dark cyan for RYR and in light purple, purple, and dark purple for VRW.

In this study, to improve the accuracy of our predictions for the Gadd45β:tripeptide complexes, we devised an MD protocol to orthogonally test the stability of different binding modes using a simple yet reliable protocol (see Figure S6e). Specifically, we clustered MD simulations at 310 K (for each of the two peptides, RYR and VRW) and utilized the Gadd45β:tripeptide complexes representative of the most populated structures to seed multiple independent high‐temperature MD simulations. Indeed, for both peptides (RYR and VRW), the top 3 binding modes were simulated in parallel at fixed high temperatures (i.e., 330 and 370 K) to qualitatively estimate the thermal stability of complexes and cross‐validate the results obtained by MD at physiological temperature (Figure 6). We opted for the high‐temperature MD simulations approach due to its lower computational resource requirements compared to conventional REMD simulations. High‐temperature MD simulations are indeed an established approach to probe the stability of protein complexes as they can accelerate conformational sampling and reveal early signs of structural destabilization that might be inaccessible at ambient temperatures (Ding et al. 2025; Pavan et al. 2022).

These studies collectively demonstrate that high‐temperature MD is a robust and widely accepted strategy to investigate the structural stability and binding properties of protein complexes. Incorporating such simulations into our analysis provides a complementary perspective and an orthogonal validation on complex stability, strengthening the robustness and generalizability of our findings. Moreover, while the primary objective of these simulations was to assess the thermal stability of the protein–peptide intermolecular contacts rather than to perform a detailed analysis of binding modes or the conformational state of Gadd45β, we nonetheless examined the protein's structural integrity throughout the simulations. Specifically, we performed secondary structure analyses for Gadd45β in complex with each pose for each peptide and at all simulated temperatures. These analyses consistently indicate that the overall folding of Gadd45β remains largely unperturbed, even at elevated temperatures (Figure S10a,b). This observation confirms that the protein maintains its native structural framework under the conditions tested, supporting the validity of the thermal stability assessments of the protein–peptide interactions, which would remain informative even in the presence of partial unfolding.

RYR:Gadd45β complexes. The high‐temperature MD results show that pose #2 of RYR is the most stable at 330 K (bound for 85% of the analyzed frames) (Figure 6a,b). Both pose #1 and pose #3 were instead observed bound for 74% of the analyzed frames. At 370 K, however, in pose #1, the tripeptide dissociates from the P1 pocket within 100 ns in each of the three replicas. Pose #2 is also the most stable RYR binding mode at 370 K, while pose #3 exhibits a similar behavior to pose #2, despite the latter's binding being globally less stable.

VWR:Gadd45β complexes. A similar trend was observed for VRW too. Here, differences in terms of % bound frames at high temperature are even more pronounced. VRW pose #3 is the most stable, resulting in bound to Gadd45β for 92% of the frames, while in pose #1 and pose #2 the peptide is bound for only 17% and 11% of the frames, respectively. When the temperature is increased to 370 K, in pose #1 and pose #2 the peptide readily dissociates from Gadd45β, remaining bound for 6% and 3% of the frames, respectively. In pose #3 it remains bound for a longer period (18%), and in one replica (r0) it stays bound for approximately 200 ns.

Based on these analyses, poses #2 and #3 of RYR and VRW, respectively, appear to be the most stable, in agreement with the results from MD simulations at physiological temperature.

Hence, high‐temperature MD simulations effectively re‐rank the heterogeneous binding modes of Gadd45β:tripeptide complexes.

We pursued an in silico mutagenesis strategy to complement our design workflow and provide mechanistic insight into peptide–protein recognition. Three mutant systems, designated αL1, αL2, and αL1–2 (Figure S11), were generated and simulated using all‐atom MD following the same protocol applied to the wild‐type systems. Each mutant was simulated in triplicate, yielding a cumulative simulation time of nearly 1 μs per system, with all simulation parameters identical to those described in section 4. Simulations were carried out at 310 K, and peptide binding was monitored by measuring the distance between the centers of mass (COMs) of the peptide and the protein residues within 6 Å of the peptide in the reference complex. Frames with COM distances greater than 6 Å were classified as unbound.

Using the most stable RYR pose (#2) as the starting structure, all three mutant systems exhibited rapid peptide dissociation, resulting in a negligible fraction of bound frames (Figure S11). These findings are consistent with our structural model, indicating that the introduced mutations disrupt critical interfacial interactions required for stable binding. While computational analyses cannot fully substitute for experimental validation the reproducibility and internal consistency of our simulations provide strong independent support for the proposed binding mechanisms.

### 2.8 Cross‐linking experiments

**FIGURE 7:**
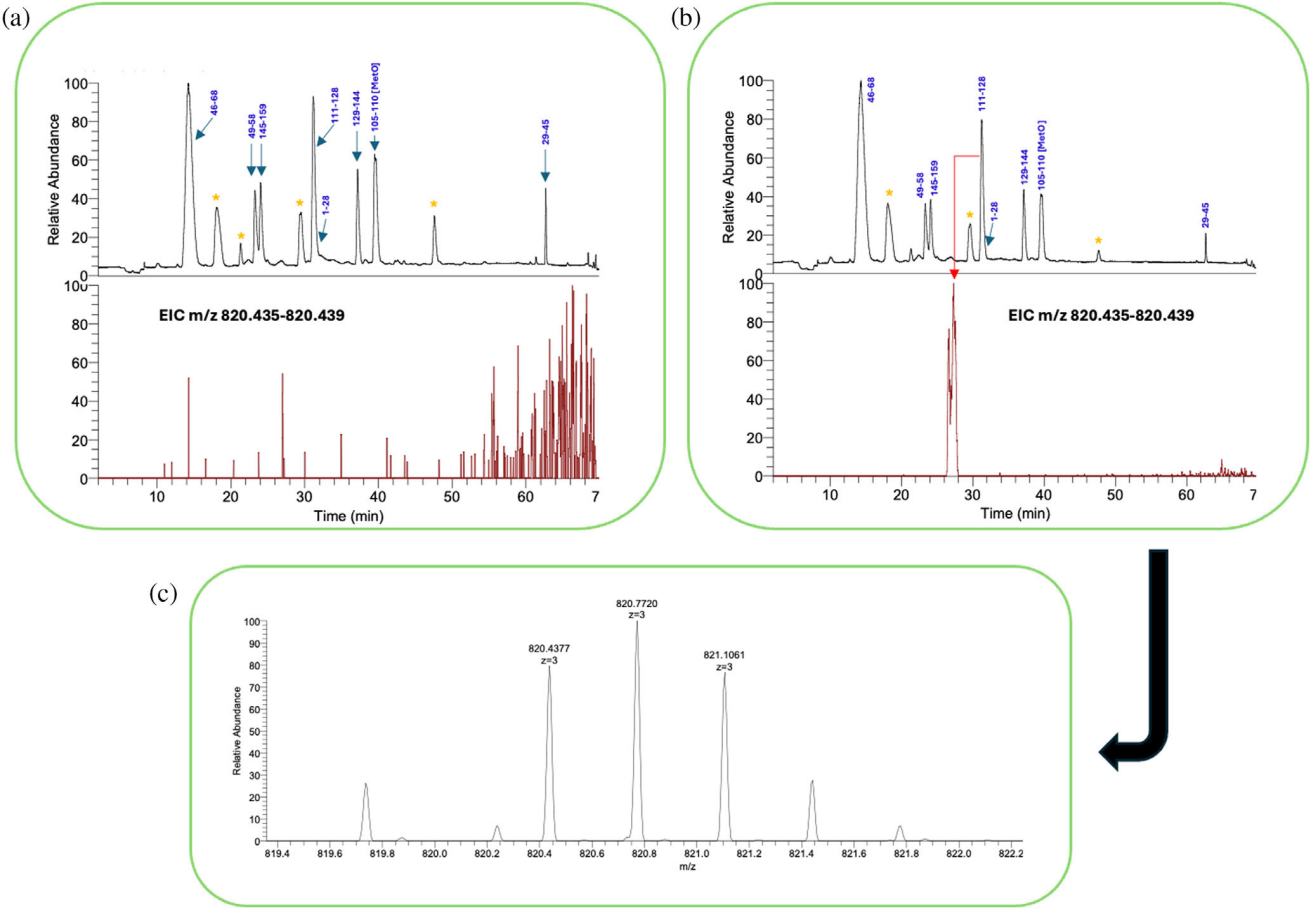
Crosslinking of RYR tripeptide to Gadd45β. (a) Upper panel: LC–MS analysis of trypsin‐digested Gadd45β not exposed to SDA‐RYR; lower panel: extracted ion chromatogram (EIC) of the expected ion at m/z 820.428 (calculated m/z 820.4315) corresponding to the first isotope of the triply charged ion of the RYR‐crosslinked Gadd45β peptide 111–128. The ion peak was identified by manually searching all possible combinations of RYR‐crosslinked peptides. (b) Upper panel: LC–MS analysis of trypsin‐digested Gadd45β exposed to SDA‐RYR; lower panel: extracted ion chromatogram (EIC) of the expected ion at m/z 820.428 corresponding to the first isotope of the triply charged ion of the RYR‐crosslinked Gadd45β peptide 111–128. Due to the high hydrophilicity of the RYR peptide, the peak is shifted at shorter retention time. The ion peak was identified by manually searching all possible combinations of RYR‐crosslinked peptides. (c) Mass spectrum of the species under the peak in lower panel of (b) showing the triply charged ion of the crosslinked peptide.

To further validate the approach, we tried to confirm the RYR peptide binding site on Gadd45β through a cross‐linking experiment. For this purpose, the peptide was N‐terminally labeled with a diazirine chemical group that can form stable covalent bonds with any electron‐donating chemical function in close vicinity to the reactive center (Rega et al. 2018). The protein was thus treated with the modified tripeptide as described in section 4, was digested with trypsin and analyzed by LC–MSMS to identify the fragments possibly crosslinked with the tripeptide. A protein sample not exposed to the tripeptide was similarly treated and comparatively analyzed under the same conditions. At first glance, no additional peaks were observed in the chromatogram of the sample exposed to the modified peptide compared to the untreated one (not shown). However, following an accurate manual search of all possible combinations of RYR‐modified Gadd45β tryptic fragments (Δmass = +574.3229 amu), we found that fragment 98–115, sequence LAQLLGEPAETQGTTEAR (MW: 1883.9591 amu) mostly corresponding to αL2, was the only one modified with the diazirine‐modified tripeptide (see Figure 7a–c).

The modified peptide, having MW 2458.2821 amu and revealed at m/z M + 3H^+^ = 820.4377 (calculated m/z 820.4351), was present at the rate of about 0.1 percent relative to the unmodified peptide, but was totally absent in the untreated protein sample. Furthermore, no other RYR‐modified Gadd45b fragments were found in the mixture, even by carefully searching for all other possible modifications that could alter the analysis, such as the presence of oxidized methionines or cysteines.

Although the low conversion rate indicates a weak recognition between the peptide and the protein under the experimental conditions reported, it unequivocally suggests that the interaction occurs in the aL2 region, as anticipated by in silico screening and corroborated by the extensive simulation data.

### 2.9 RYR tripeptide reduces cell survival in acute myeloid leukemia cell over‐expressing Gadd45β

**FIGURE 8:**
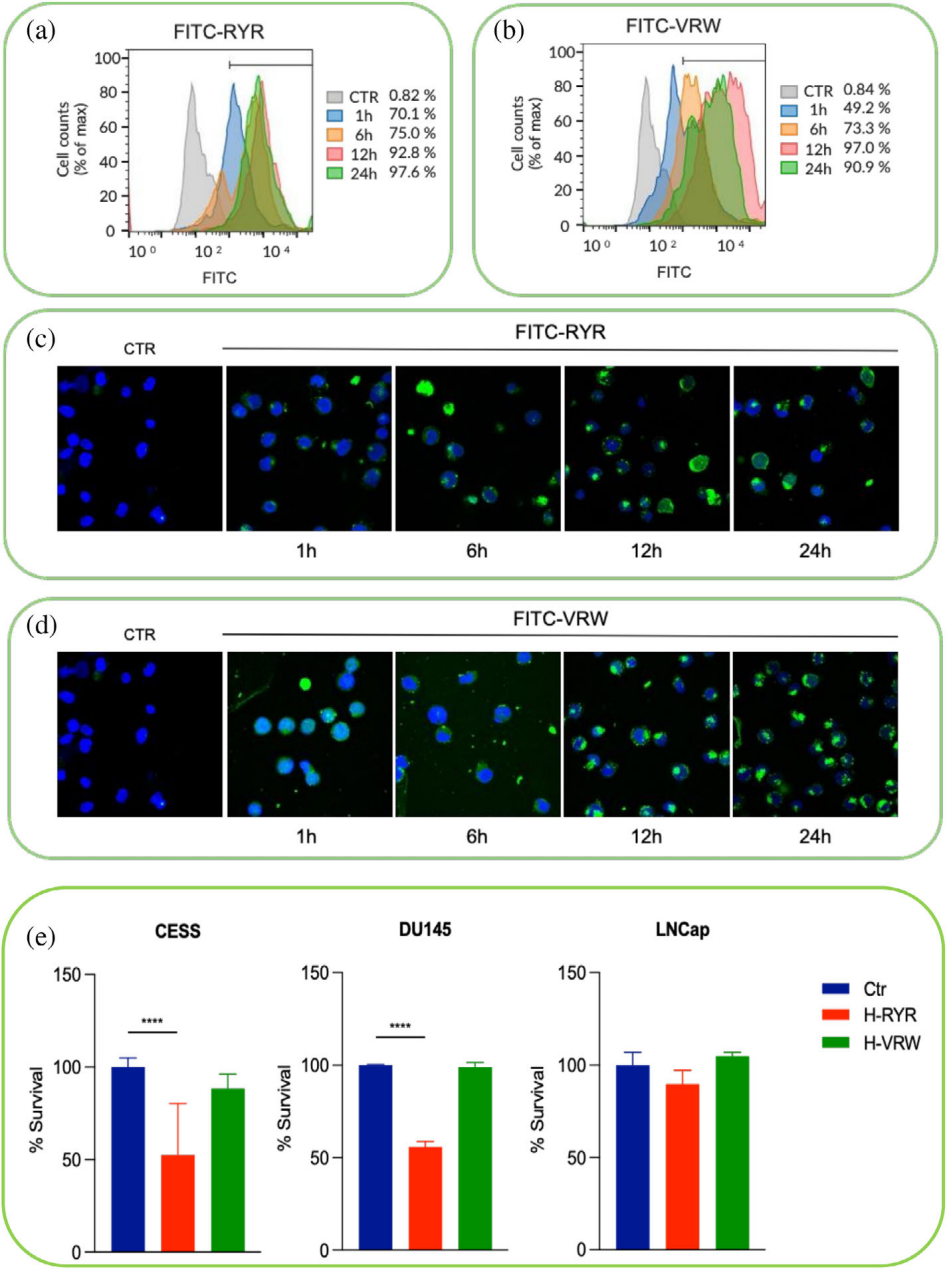
Histogram plot from FACS analysis of (a) βa‐RYR‐FITC and (b) βa‐RYR‐FITC uptake in CESS cell line at the indicated time points. Representative confocal images of immunofluorescence staining showing CESS cell line treated with (c) βa‐RYR‐FITC, (d) βa‐VRW‐FITC at the indicated time points. Green, βa‐RYR, and βa‐VRW; blue, DAPI. Magnification 40×. (e) CellTiter Glo cell viability assay showing that just RYR (not VRW peptide) reduces cell viability of CESS and DU145 cell lines after 5‐day treatment with 500 μM of RYR. Values denote means ± SD. The experiments were performed in triplicate. Statistical Analyses were performed using two‐tailed Student's *t* test. ****p < 0.0001.

Given the relevance of cavity P1 for the protein biological activity, we next investigated the impact of administering the P1‐interacting tripeptides to a set of cancer cells that express different levels of Gadd45β: CESS, DU145, and LNCaP. In a preliminary experiment, using FACS and immunofluorescence, we evaluated the ability of the two tripeptides RYR and VWR, opportunely modified at the N‐terminus with FITC, to penetrate the cell membrane of acute myeloid leukemia CESS cells. Following treatment with the two FITC‐tripeptides, we observed a time‐dependent uptake for both molecules (Figure 8a–d), although at high concentration (500 μM). Next, to test the efficacy of peptides in inducing cell death, we treated a panel of three cancer cell lines expressing high (CESS; DU145) or low (LNCaP) levels of Gadd45β with either RYR or VRW (Figure S12). As shown in Figure 8e the treatment with RYR reduced the viability of CESS and DU145 cells while the same was ineffective on LNCaP cells expressing only low levels of the target protein. Conversely, treatment with VRW did not affect cell viability of any of the analyzed cell lines (see Figure 8e) suggesting that while RYR preserved its selective activity on cells expressing high levels of Gadd45β the other peptide was not.

These results, although partially discordant with the binding data, suggest that at least one of the peptides is also active in cells and is therefore a promising hit to generate new, more potent compounds capable of targeting the P1 cavity of Gadd45β, which is strongly implicated in cancer development. The failure of VRW is likely ascribed to the combined effect of the lower quantity that enters the cells, compared to the RYR peptide, and its different intracellular fate. VWR indeed, due to its greater hydrophobicity, could be sequestered unproductively within the cell by binding to membranes, or could leak out rapidly, thus reducing its concentration and resulting in biologically ineffective outcomes. Collectively, the concurrent use of in silico and wet approaches has enabled the selection, from a quite large set, of a couple of D‐tripeptides that bind Gadd45β on a specific and biologically relevant protein site, highlighting the impact of combining multiple approaches in early drug discovery phases. A great contribution has been obtained from computational analyses which have made possible the initial screening in silico of hundreds of different D‐tripeptide combinations, reducing at the same time the synthesis and wet bench assays work. Such combined approaches will play an increasingly important role in guiding the design and development of future sets of small ligands for Gadd45β, as well as other relevant therapeutic targets, that can be further optimized toward more effective and potent molecules.

## 3 CONCLUSIONS

We have here reported an integrated approach that combines a screening software (Cross et al. 2010), solid‐phase synthesis of very short peptides, label‐free screening of a selected set of molecules, STD‐NMR, advanced computational simulations (like ensemble docking or molecular dynamics), and cell‐based assays.

Application of this approach has led to the quick definition of the structural determinants necessary for the molecular recognition of active sites of proteins of therapeutic interest.

We have chosen as an example of the target protein Gadd5β which has peculiar structural features with well‐studied regions and active sites (Capece et al. 2018; Papa et al. 2004a; Papa et al. 2004b; Papa et al. 2008; Rajpoot et al. 2020; Sandomenico et al. 2020; Tornatore et al. 2014b; Verzella et al. 2018; Zazzeroni et al. 2003). As a source of binding structures, we have chosen the chemical space described by tripeptides in D configuration to obtain protease‐resistant molecules having few functional groups, so that they can be more easily converted into small molecules. On the peptides selected through a label‐free binding assay we have conducted an extensive STD‐NMR characterization to define the functional groups involved in the recognition and an extensive in silico structural characterization of the molecular complexes formed by two of the selected tripeptides as binders for a cavity of the protein involved in important biological functions.

Despite the relatively low affinity achieved (μM range), the data obtained are very comforting because we have rapidly obtained, with a limited synthetic and wet screening effort, specific binders for predefined binding sites. The structural data indeed indicate that a wide network of interactions, which involve aromatic and aliphatic groups in addition to polar and ionic sites, underpins the recognition between the protein and the two hits.

This approach, which can be optimized using other in silico screening platforms (Vincenzi et al. 2024) or other more performing parallel wet screening methods (Bruce et al. 2024; De Keyser et al. 2025), outlines a proposal for a minimal combination of complementary technologies needed to identify molecular precursors of hit compounds for specific protein cavities. The approach requires limited synthetic, screening and structural investigation efforts and can lead to molecules of broad application, especially PPIs, the most interesting and difficult‐to‐study class of active molecules.

**FIGURE 9:**
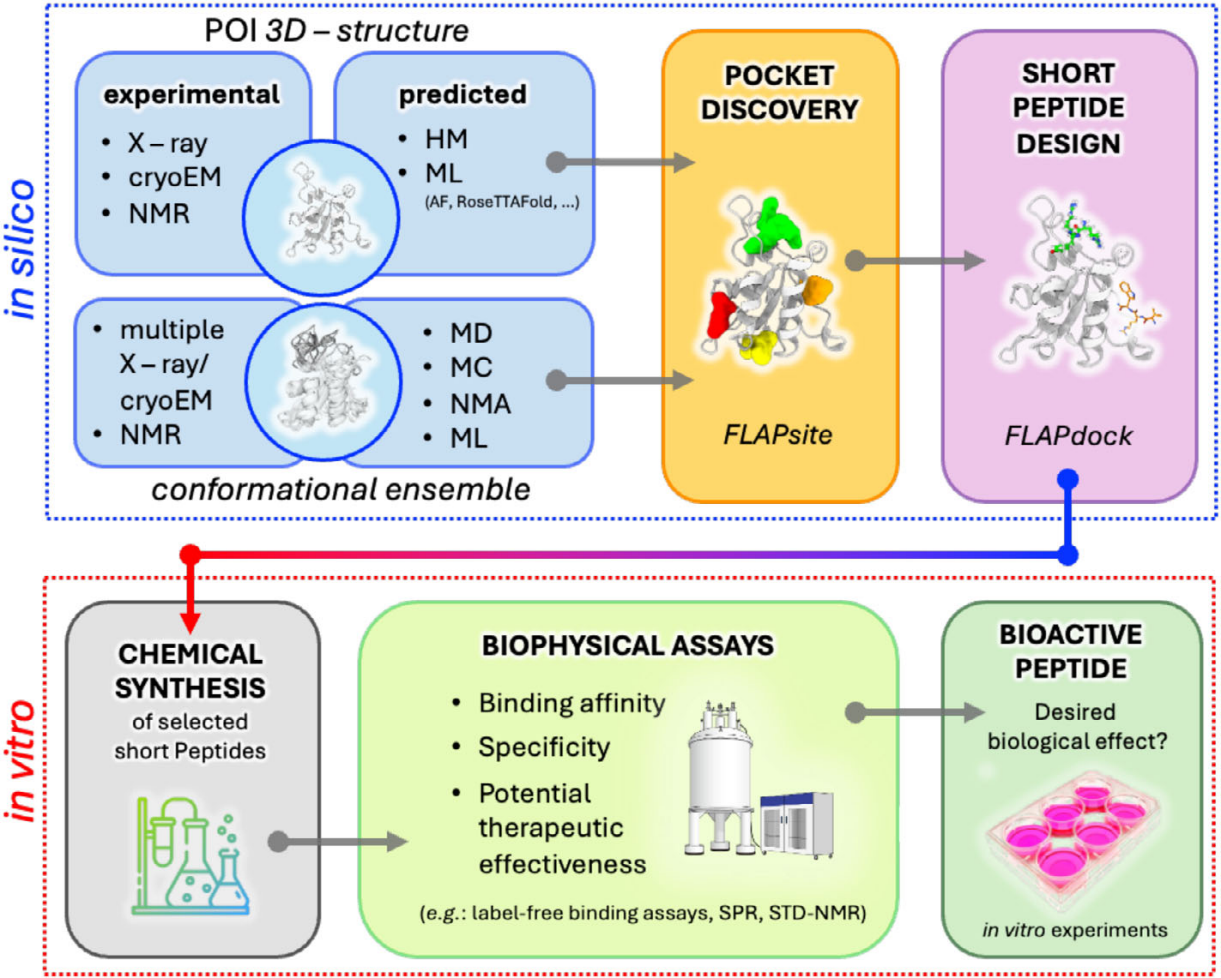
Computational–experimental synergistic workflow for the design and validation of short peptides. Structural preparation of the protein of interest (POI) precedes peptide design. The POI structure can be obtained experimentally (X‐ray crystallography, NMR, cryo‐EM) or computationally through homology modeling (HM) or machine learning (ML)–based prediction (e.g., AlphaFold, RoseTTAFold). When experimental data are unavailable, molecular dynamics (MD), Monte Carlo (MC), Normal Mode Analysis (NMA), or ML‐based conformational sampling can generate relevant conformers capturing backbone and side‐chain flexibility. Prepared structures are processed through FLAPsite and FLAPdock to design peptides based on chemico‐physical pocket features. The in silico stage yields putative peptide binders and approximate binding modes, which can be refined by atomistic simulations (see Figure S6). Designed peptides are then synthesized and experimentally validated using biophysical assays (e.g., SPR, STD‐NMR, label‐free binding). Promising candidates are tested in vitro to confirm biological activity and potential therapeutic effects.

The approach, which can be extended to other therapeutic targets, must be seen as a tool to rapidly access new hit compounds that can be modified and optimized to match the pharmacodynamic and pharmacokinetic properties peculiar to small molecules and thus converted into possible drug candidates (Figure 9).

## 4 MATERIALS AND METHODS

### 4.1 GADD45β 3D structure modeling

To date, the experimentally determined three‐dimensional (3D) structure of Gadd45β is not available. Wild‐type Gadd45β protein structure was predicted using a local installation of AlphaFold2 software. The source code was obtained from the AlphaFold2 Github page (https://github.com/deepmind/alphafold). The primary sequence of Gadd45β was retrieved from the UniProt database (ID: O75293) and used as input. We employed the full genetic database configuration and included a final relaxation step on all predicted models. The AlphaFold2 structure prediction workflow predicted five structures, starting with a random seed and ranked by their predicted local difference distance test (pLDDT) score on a scale from 0 to 100. The top‐ranked model was selected as the final one and subjected to relaxation using the Rosetta FastRelax protocol (Tyka et al. 2011) for subsequent optimization. We used the pLDDT parameter to measure which regions of the protein were predicted with high confidence. On this basis, the N‐terminal tail (residues 1–15) was discarded due to a predicted pLDDT score lower than 70. Also, acidic loop 2 (residues 103–117) exhibited a pLDDT score lower than 70; hence, it was rebuilt de novo by means of Rosetta Next‐Generation KIC (Stein and Kortemme 2013). A total of 1000 loop models were generated and ranked based on the “*total_score*” Rosetta energy term. Following loop building, the top‐ranked model underwent thorough relaxation using the Rosetta full‐atom FastRelax protocol. The REF2015 Rosetta energy function was utilized for all experiments (Park et al. 2016).

### 4.2 Gadd45β binding pockets identification

The identification of Gadd45β cavities was conducted using FLAP (Fingerprints for Ligands And Proteins) software, developed and licensed by Molecular Discovery Ltd. (www.moldiscovery.com) (Baroni et al. 2007; Cross et al. 2010). FLAP utilizes Molecular Interaction Fields (MIFs) generated via the GRID force‐field (Carosati et al. 2004) to assess the type, strength, and direction of the interactions that a molecule can establish. The fingerprints are condensed into discrete points and exhaustively combined to produce quadruplets of pharmacophoric features. They can be used for ligand–ligand, ligand–receptor, and receptor–receptor comparison.

Gadd45β atomic coordinates were imported into FLAP by applying the predefined FLAP base filters for .*pdb* files. The FLAP*site* algorithm was used for the identification of cavities in the protein 3D structure (Henrich et al. 2010). FLAP*site* starts by embedding the protein structure into a three‐dimensional grid with a spatial resolution of 1.0 Å. The algorithm identifies pocket points using GRID probe H (hydrogen probe H is used to compute protein cavity shape). Subsequently, protein surface points are excluded, and a buriedness index is calculated for the remaining points. Points with a buriedness index lower than a specific threshold are discarded. Morphological operations, including erosion, and dilation, are then applied to the remaining points for removing small anomalies and connecting areas (Haralick et al. 1987). FLAP prioritizes hydrophobic sites, typically targeted by drugs, using the GRID hydrophobic probe (DRY) to identify places where hydrophobic atoms on the surface of a target molecule can interact favorably with hydrophobic ligand atoms.

### 4.3 Computational design of linear tripeptides

D‐tripeptides design was implemented through an approach akin to standard virtual screening methods. Initially, a virtual library of all combinations of linear D‐tripeptides was built. This library was screened with a version of FLAP*dock* software, the docking tool implemented in the software FLAP optimized for peptide docking (Baroni et al. 2007; Cross et al. 2012). To begin, we have used all combinations of tripeptides with 19 of the 20 standard D‐amino acids (excluding cysteine) to generate an ensemble of 19^3^ = 6859 single tripeptides as potential interactors of GADD45β on 4 specific cavities. This library underwent screening with FLAP software (Baroni et al. 2007; Cross et al. 2010) to perform fast virtual screening in a receptor‐based pharmacophore approach. The screening involved matching 50 diverse conformations of each peptide to the GRID MIFs (Carosati et al. 2004) of the Gadd45β cavities, previously identified by FLAP*site*. The top 1% of the ranked tripeptides were subsequently docked into the identified cavities using a modified version of FLAP*dock* software (Baroni et al. 2007; Siragusa et al. 2016). This version of FLAP*dock* software splits molecules into small fragments and docks each fragment into the binding site by matching pharmacophoric quadruplets in the fragment and the receptor site GRID MIF. The fragments are scored based on their GRID MIF overlap, considering FLAP field similarities, including shape, donor, acceptor, and hydrophobic similarity as well as Lennard‐Jones, Coulombic, and desolvation terms. The top scoring poses were then used to grow the remaining fragments at varying dihedral angles, sampling torsional space to reconstruct the ligand. At each stage, solutions were scored and filtered until final poses of the complete ligand were minimized, rescored, and ranked in the cavity. For peptide docking, the fragments are consistently generated by cutting the bonds on either side of the backbone amide, with the amide fragments excluded as starting positions. Conformations of the remaining fragments were generated using a modified version of the MM3 force field (Lii and Allinger 1991; Vanommeslaeghe et al. 2014) introducing parameters tailored for peptides. The top 100 best‐scored tripeptides for every cavity were extracted, and from these, the best 8 targeting single Gadd45β cavities were selected for chemical synthesis and further biophysical and computational screening.

### 4.4 Peptide synthesis procedures and preparation of Gadd45β recombinant protein

Peptides synthesis involved the generation of tripeptides with C‐terminal amides in the D configuration and free N‐termini to ensure protease‐stable molecules and with an additional positive charge to enhance solubility. The assembly of fully protected peptides was manually conducted in polypropylene Grace™ reaction vessels using the Fmoc solid‐phase strategy on a synthesis scale of 50 μmol (Caporale et al. 2017; Caporale et al. 2018). The synthesis was performed on a RINK amide resin with a nominal loading of 0.72 mmol/g. Standard Fmoc‐protected amino acids were used for all synthesis. The Fmoc protecting group was removed by treatment with piperidine 40% in DMF for 5 min and piperidine 20% in DMF for 10 min. Coupling reactions were performed using 4 eq. of Fmoc‐protected derivatives activated in situ with HATU (4 eq.) and collidine (8 eq.) for 45 min (Caporale et al. 2017; Caporale et al. 2018). The coupling efficiency was assessed by the Kaiser test after each coupling (Sarin et al. 1981). Each step was followed by resin washing (3 × 5 min) with 1 mL DMF. The fluoresceine isothiocyanate (FITC)‐labeled variants (FITC‐βAla2‐VWR and FITC‐βAla_2_‐RYR) were similarly assembled on the solid phase introducing on the N‐terminus two additional β‐alanines and then FITC at the N‐terminus via direct overnight coupling (room temperature) to the resin. FITC was dissolved in DMF and used at 5‐fold excess at slightly basic pH. Peptides were deprotected and cleaved from the resins by treatment with a mixture of TFA/H_2_O/TIS (90:5:5 v/v/v) for 3 h. Resins were removed by filtration and the crude peptides precipitated by adding cold diethyl ether. The precipitates were recovered after centrifugation and lyophilized after dissolution in 50:50 H_2_O/CH_3_CN. Peptides were purified to homogeneity by semi‐preparative RP‐HPLC using a XBRIDGE Prep BEH130 C18, 5 μm OBD™ 19 × 50 mm column equilibrated at 20 mL/min with 5% CH_3_CN, 0.1% TFA (Solvent B; Solvent A was H_2_O, 0.1% TFA). To elute the peptides gradients from 5% to 40%–80% Solvent B were applied, depending on peptide hydrophobicity. After lyophilization, the purified molecules were characterized by LC–MS in terms of purity and identity and stored frozen until use. Polypropylene reaction vessels endowed with filtration septa were supplied by Grace™. RINK amide resin, Wang resin, coupling reagents, and all amino acids were purchased from IRIS Biotech‐GmbH (Marktredwitz, Germany). Collidine, piperidine, TFA, TIS were supplied by Sigma Aldrich (Milano, Italy); DMF, DCM, and Et_2_O were from Romil (Dublin, Ireland). HPLC analyses were performed on a Waters Alliance e2695 supplied with a 2998 PDA detector and equipped. Preparative separations were performed using a HPLC Waters 2545 Quaternary Gradient Module supplied with a Waters 2489 UV/visible Detector and equipped with a preparative XBRIDGE Prep BEH130 C18 5 μm OBD™ 19 × 50 mm column (Milano, Italy). Mass spectrometric analyses were carried out on an Agilent ESI‐TOF LC–MS system comprising an Agilent 1290 Infinity LC coupled to an Agilent 6230 TOF mass spectrometer. A Xbridge C18 50 × 2 mm ID column from Waters was used for all analyses applying a gradient from 5% solvent B to 60% solvent B in 20 min. Solvent A was H_2_O, 0.08% TFA; solvent B was CH_3_CN, 0.05% TFA. Flow rate was 0.2 mL/min. Detection in positive ion mode was between m/z 300 and 3000. After lyophilization, the purified molecules were characterized by LC–MS in terms of purity and identity and stored frozen until use. Recombinant human Gadd45β was prepared by expression in *E. coli* and purified as previously described (Papa et al. 2007; Rega et al. 2018; Tornatore et al. 2014a).

### 4.5 Surface Plasmon resonance experiments

To determine the binding affinity constant of the selected D‐tripeptides for the human recombinant Gadd45β protein, real‐time binding assays were conducted using the surface Plasmon resonance (SPR) technique on a Biacore 3000 instrument (GE Healthcare, Milan, Italy). In summary, Gadd45β was covalently immobilized onto the dextran matrix of a CM5 sensor chip through the primary amine groups using an amine coupling kit (GE Healthcare). The carboxymethylated dextran surface was activated by injecting a mixture of 0.2M N‐ethyl‐N′‐(diethylamino‐propyl)carbodiimide and 0.05M N‐hydroxysuccinimide (EDC/NHS chemistry) in accordance with the manufacturer's instructions (Johnsson et al. 1991).

The ligand immobilization process was efficiently carried out at 50 μg/mL in 10 mM sodium acetate buffer pH 3. Residual N‐hydroxysuccinimide esters were blocked by exposure to 1M ethanolamine hydrochloride (pH 8.5). A reference channel was prepared and utilized as a control blank. All immobilization steps were conducted at a flow rate of 5 μL/min using 10 mM HEPES, 150 mM NaCl, 3 mM EDTA, 0.005% P20 (HBS‐EP, GE Healthcare) (pH 7.4) as the running buffer. Selected D‐tripeptides were injected at concentrations ranging from 25 to 200 μM. Where needed, after analyte injection the surface was regenerated with pulses of a 5 mM NaOH solution. Analyses were performed at 25°C at a flow rate of 20 μL/min in HBS‐EP buffer (GE Healthcare). In all binding experiments, association phases were monitored for 180 s, and dissociation phases for 300 s. Non‐specific binding from the reference channel was subtracted from the working channels using the BIAevaluation analysis package (version 4.1, GE Healthcare). Data fitting was conducted using GraphPad Prism software (GraphPad Software, San Diego, CA).

#### 4.5.1 NMR spectroscopy

1D, Saturation Transfer Difference (STD), and 2D NMR spectra were recorded at 299 K using an Agilent Varian Inova spectrometer operating at a proton frequency of 600 MHz equipped with a 5‐mm inverse‐detection cryo‐probe and Z‐gradient, located at the Istituto di Biostrutture e Bioimmagini (IBB) of CNR, Napoli, Italy.

Saturation‐transfer difference (STD) NMR experiment (Mayer and Meyer 1999; Mayer and Meyer 2001) is obtained by selectively saturating the protein resonances and observing which ligand protons undergo signal variation due to saturation transfer. In such a way the ligand's atoms interacting with the target protein are identified. STD involves the acquisition of 2 one‐dimensional experiments “off‐resonance” and “on‐resonance” spectra, on a sample containing both the protein at low μM concentration and the ligand in a large excess. The “off” and “on” spectra are acquired in the first case by irradiating a region far from both protein and ligand signals (at 20–40 ppm) and in the second case by irradiating a region containing exclusively protein signals. Irradiation is obtained by using a train of radio frequency pulses centered at “off” or “on” frequencies. The prolonged irradiation in the “on” spectrum produces spreading of saturation to the protein protons and saturation transfer to the ligand protons in contact with the protein. The STD experiment, which is obtained by subtracting the “on‐” and “off‐resonance” spectra, reveals only signals belonging to ligand protons at short distances from the protein. To find the right frequency for “on resonance” spectra, both protein and peptides were separately characterized by NMR. The free peptides were analyzed in plain water (1 mM in H2O/D_2_O 90:10, v: v). Gadd45β was analyzed at a concentration of 210 μM in deuterated TRIS buffer 20 mM/D_2_O at pH 7.5 (not corrected for D_2_O), with NaCl 50 mM and deuterated TCEP (tris(2‐carboxyethyl)phosphine) 0.5 mM. D_2_O was purchased from Sigma, while TCEP and TRIS were obtained from Cambridge Isotope Laboratories.

In our STD measurements, a 50 ms Gaussian pulse‐train at 0 ppm (−3045 Hz) or 5.2 ppm (146 Hz) for “on‐resonance” spectra, and at 27 ppm for all “off‐resonance” ones was used. The magnetization was allowed to spread for a time typically of 2 s. Excitation sculpting pulse sequences were employed to suppress the water signal in the spectra.

The binding between each protein/peptide couple was assessed by acquiring a series of STD experiments at molar peptide/protein ratio, R, of 10, 20, 30, 50, 70, 100, and 200. The desired R values were achieved by adding convenient peptide micro‐volumes to protein sample at a fixed amount (initially 20 μM, see above for solvent conditions). The spectra were processed and analyzed using MESTRENOVA 6.0 software (Mestrelab Research, S.L, Santiago de Compostela, Spain).

The proton resonances of the peptides in the presence of protein were assigned at a peptide/protein ratio equal to R100 using standard 2D sequences as TOCSY, NOESY, and ROESY. The data file typically comprised 512 and 4096 data points in the w1 and w2 dimensions, respectively. TOCSY experiments were acquired with a 70 ms mixing time, NOESY experiments with a 400 ms mixing time and ROESY experiments with a 300 ms mixing time. To delineate the ligand group epitope mapping (GEM), STD effects were quantified by integrating the peak areas at a peptide/protein ratio of R100 and normalizing to the highest intensity peak, set at 100%.

### 4.6 Ensemble molecular docking between Gadd45β and peptides

We employed an ensemble docking approach corresponding to the generation of an “ensemble” of Gadd45β conformations, thereby accommodating the flexibility of the receptor protein. To characterize protein dynamics, molecular dynamics (MD) simulations were conducted. Several thus‐generated conformations of the protein were then used in multiple docking calculations, as opposed to docking to a single structure of the target protein. The conformational ensemble here selected consisted of the 10 representative structures that best capture the flexibility of Gadd45β acidic loop 2, obtained upon MD trajectory clustering (see below).

D‐tripeptides were built in silico as C‐terminal amides in the D‐configuration with free N‐termini using Avogadro 1.2.0 software (http://avogadro.cc/), protonated at pH = 7.4 and energy minimized using the GAFF force field until dE ~ 0. An ensemble docking campaign was performed using two different software packages, namely AutoDock VINA (Trott and Olson 2009), implementing a stochastic global optimization approach, and the recently developed GNINA program (https://github.com/gnina/gnina) (McNutt et al. 2021), whose scoring function is based on an ensemble of convolutional neural networks (CNNs). The GNINA CNN models output both a CNNscore and a CNNaffinity for each of the conformations output by GNINA. The CNNscore is a value between 0 and 1 and it is used to rank the poses of the ligand. CNNaffinity is the affinity of the docked complex as determined by the CNN. This metric has been evaluated in a previous work (McNutt et al. 2021). The default settings were used in all cases, except for the exhaustiveness parameter (giving a measure of the exhaustiveness of the local search), which was increased to 1024 (the default value is 8). The grid box was set to wrap around the cavity of the Gadd45β to be explored (pocket_1), comprising acidic loop1, acidic loop2, and part of H1, H2, and H3 helices (extending for 6 Å around the ligand in each dimension).

Docking calculations using the ensemble of conformations generate a total of 90 binding modes for every Gadd45β:peptide complex. Based on STD‐NMR results we defined a “*SASA_filter*”: we quantitatively measured the solvent‐accessible surface area (SASA) values of aromatic residues on the D‐tripeptides after engaged by Gadd45β to discard docking poses that exhibited solvent‐accessible surface area for Tyrosine or Tryptophan side chain greater than 20 Å^2^. In this way, only poses with aromatic residues penetrating deeper in the binding cavity were retained for subsequent analysis. To increase Gadd45β:peptide complex prediction accuracy, we used three different scoring functions (Vina, CNNscore, and CNNaffinity) to rank the filtered poses (according to “*SASA_filter*”).

Thus, for each protein:peptide complex, we did not use a consensus ranking. We picked the top‐ranked pose according to the Vina scoring function (that we identified as pose #1), the top‐ranked pose according to CNN_score (identified as pose #2) and the top‐ranked pose according to CNN_affinity score (identified as pose #3) as the most promising binding modes. Then these (three) selected poses (pose #1, pose #2, and pose #3 for each protein:peptide complex) were extensively simulated by means of multiple independent μs‐long MD starting from 310 K to increasing temperatures (as detailed below), with the aim to select the final binding pose per peptide.

### 4.7 Molecular dynamics simulations and cluster analysis

All‐atom multiple independent μs‐long molecular dynamics (MD) simulations in explicit solvent were performed with GROMACS 2020.4 code (https://zenodo.org/records/4054996). For both unbound Gadd45β and Gadd45β:peptide complex models the same steps were performed with a few deviations noted below. In all cases the protein was described by the Amber‐99SB‐ILDN force field (Lindorff‐Larsen et al. 2010). The system was then solvated in a cubic box of TIP4P water (Lindorff‐Larsen et al. 2010), with 12 Å of padding around the solute in each dimension. To reduce the charge to neutral and to match the 0.15M NaCl solution used in the experimental buffer, Na^+^ and Cl^−^ ions were added. An integration time step of 2 fs was used, and all covalent bonds involving the hydrogen atoms were constrained with the LINCS algorithm. We used the Particle Mesh Ewald scheme to account for the electrostatic interactions using a real space cut‐off of 10 Å. Production MD simulations were performed in the isothermal–isobaric NPT ensemble at a temperature of 310 K and under the control of a velocity‐rescaling thermostat, where protein and non‐protein atoms were coupled separately to a temperature bath. A Parrinello–Rahman barostat was used to maintain an isotropic pressure of 1 bar. For each system, preliminary energy minimization was performed by employing 50.000 steps of the steepest descent algorithm. Equilibration was first performed in the NVT ensemble at the temperature of 310 K using the velocity‐rescaling thermostat, then we switched to the NPT ensemble, scaling the pressure to 1 bar using the Parrinello–Rahman barostat, keeping the entire system restrained except for the solvent atoms and the solute hydrogens. Finally, unbound Gadd45β protein was simulated for 1 μs, whereas each protein:peptide system was simulated for a minimum sampling time of 0.9 μs (more than 300 ns × 3 replica) of unbiased MD. In the case of protein:peptide complexes, the trajectories at *T* = 310 K were used as a starting point for high‐temperature unbiased MD simulations. That is, for each simulated complex, a representative structure was extracted (see *Clustering analysis* paragraph below) from the concatenated trajectories at *T* = 310 K and the same structure was used to seed high‐temperature MD at *T* = 330 K and *T* = 370 K. Details about all simulated systems are reported in Table S1. Unless otherwise stated, all simulation analyses were conducted with the *cpptraj* module (Roe and Cheatham 2013) in AmberTools2022 (Case et al. 2023), GROMACS 2020.4 analysis tools (https://doi.org/10.5281/zenodo.3562495) and in‐house python code using *numpy* and *scipy* packages.

When we performed high‐temperature MD, we measured the distance between the center of mass (COM) of the peptide and the COM of the binding site (i.e., residues within a radius of 5 Å from the peptide) to define whether the peptide detached from the binding site. We set a cut‐off equal to 6 Å above which the peptide was considered unbound. For the calculation of the protein's center of mass (COM), we considered only the Cα atoms of residues located within a 6 Å radius from the specific binding mode of each peptide. Consequently, the set of residues included in the COM calculation varies depending on the precise binding pose adopted in each simulation. This approach ensures that the COM accurately reflects the local environment of the bound peptide while maintaining consistency in the relative definition of the binding site across different starting structures. Volumetric maps of D‐tripeptides distribution around Gadd45β were obtained using the “grid” command of *cpptraj* with a grid spacing of 0.5 Å. Average structures used for comparison between different complexes were obtained by doing an RMS mass‐weighted fit to the initial structure followed by a straight coordinate average over all frames.

Clustering of the unbound Gadd45β MD trajectory was carried out using two different clustering algorithms implemented in *cpptraj* (Roe and Cheatham 2013): (i) the average‐linkage hierarchical agglomerative clustering method employing an RMSD cut‐off of 2.5 Å, calculated on all the heavy atoms of the acidic loop 2 backbone (aa 103–117), (ii) the *K*‐means clustering algorithm on all the heavy atoms of the acidic loop 2 backbone (aa 103–117), setting the number of clusters to 10. In comparison to hierarchical agglomerative clustering, the *K*‐means algorithm yielded the best results based on the Davies–Bouldin Index (DBI) and psF (clustering quality metrics) and exhibited a higher value of R‐squared (percentage of variance explained by the data).

Thus, we used 10 representative structures obtained from *K*‐means approach to consider the acidic loop 2 flexibility in subsequent ensemble docking.

Clustering of Gadd45β:peptide complexes MD trajectories at *T* = 310 K was performed using the *K*‐means algorithm on acidic loop 1 (residues 62–68), acidic loop 2 (residues 103–117), and D‐tripeptides heavy atoms, obtaining 10 representative structures for each Gadd45β:peptide complex. The representative structure of the most populated cluster was selected as a starting point to seed high temperature MD simulations at *T* = 330 K and *T* = 370 K. The simulation temperatures (330 and 370 K) were chosen following a systematic and reproducible strategy, while remaining below a safety threshold of 100°C (373 K), a temperature at which many proteins denature. The initial temperature of 310 K represents standard physiological conditions. To explore progressively more extreme conditions in a controlled manner, we applied temperature increments of differing magnitude: a first “soft” increment of +20 K to reach 330 K, followed by a slightly larger increment of +40 K to reach 370 K. A detailed contact analysis was carried out using the script *get_contact* (https://getcontacts.github.io/) that calculates different types of residue contacts including hydrogen bond, van der Waals, salt bridge and cation–π contacts. A pair of residues was considered to form a sustained contact during the simulation if the contact frequency was greater than 25%.

### 4.8 Molecular mechanics/generalized born surface area

For molecular mechanics/generalized born surface area (MM/GBSA) binding free energy estimation we defined a set of uncorrelated snapshots that adequately sample the equilibrium ensemble and at the same time ensure the computational treatability of the calculation. Indeed, we down‐sampled our 310 K MD trajectories as follows: for Gadd45β:RYR complex MD simulations at *T* = 310 K, we merged 250–300 ns trajectories from three independent simulations for each pose. Subsequently, we analyzed the merged trajectories resulting in 7500 frames/pose. For Gadd45β:VRW complex MD simulations at *T* = 310 K, we merged 0–300 ns trajectories from three independent simulations for each pose and then analyzed the merged trajectories (7500 frames/pose).

Each Gadd45β:peptide complex MM/GBSA relative binding free energy was computed with the *mmpbsa.py* module of AmberTools22 (Case et al. 2023). gmx‐MMPBSA (v1.5.1) (Valdés‐Tresanco et al. 2021) was used to convert the GROMACS trajectories into AMBER format with ParmEd and subsequently used *mmpbsa.py*. Energies were computed with a surface tension of 0.0072 kcal/mol/Å^2^. The non‐polar contribution to the solvation free energy was approximated using the LCPO method (Huang and Simmerling 2018). A single trajectory MM/GBSA protocol was adopted to calculate the binding free energy differences, neglecting the solute entropic contribution. Since the ΔG_binding_ was calculated by omitting the entropic term it is referred to as relative binding energy. Solvent‐accessible surface area (SASA) was calculated with visual molecular dynamics (VMD 1.9.3) built‐in tools (https://www.ks.uiuc.edu/Research/vmd/) (Humphrey et al. 1996).

### 4.9 Cross‐linking of the Gadd45β/D‐tripeptide complexes

#### 4.9.1 Cross‐linking of peptide RYR to GADD45β

To perform these experiments the tripeptide RYR labeled with a photoactivable diazirine reagent (NHS‐SDA; Pierce Biotechnology, ThermoFisher), followed an approach previously described to assess the binding sites of DTP3 and MKK7 on GADD45β. For this purpose, the tripeptide was modified at its N‐terminus with NHS‐preactivated reagent as previously reported (Rega et al. 2018) and purified to homogeneity. For the photo‐labelling step, GADD45β (1.2 nmol, 20 μM in sodium phosphate buffer pH 8.0) was incubated for 15 min at room temperature in the dark with a 15‐fold excess (mol/mol) of SDA‐labeled peptide. Equal amounts of GADD45β without the labeled peptides were analyzed in parallel to evaluate the specificity of the reaction.

For the UV‐cross‐linking reaction (60 μL final volume) the diazirine group was photo‐activated by UV irradiation at 365 nm for 15 min on ice as described elsewhere (Rega et al. 2018). Removal of unreacted SDA‐labeled peptides and buffer exchange to 50 mM ammonium bicarbonate pH 7.8 was performed using Corning Spin‐X UF Concentrators MWCO 5000 (Millipore).

#### 4.9.2 In solution tryptic digestion

Cross‐linked samples were reduced at 55°C for 1 h by adding dithiothreitol (DTT) up to 10 mM final concentration. Following carbamidomethylation with iodoacetamide (IAM, 7.5 mM final concentration) at room temperature in the dark for 15 min, enzymatic hydrolyses were performed with TPCK‐treated bovine trypsin (Sigma Aldrich, Milan, Italy) at 37°C for 16 h with an enzyme‐to‐substrate ratio of 1:50 (w/w). After digestions, samples were centrifuged at 10,000*g* for 15 min and supernatants were diluted in H_2_O/Formic acid 0.1% at a final concentration of 500 fmol/μL for MS analysis.

#### 4.9.3 Identification of cross‐linked products by high‐resolution nanoLC‐tandem mass spectrometry

Cross‐linked samples prepared as described above, were analyzed by high‐resolution nanoLC–tandem mass spectrometry using a Q Exactive Orbitrap mass spectrometer equipped with an EASY‐Spray nano‐electrospray ion source (Thermo Fisher Scientific) and coupled to a Thermo Scientific Dionex UltiMate 3000RSLC nano system (Thermo Fisher Scientific). Solvent composition was 0.1% formic acid in water (solvent A) and 0.1% formic acid in acetonitrile (solvent B). Peptides were loaded on a trapping PepMap™100μ Cartridge Column C18 (300 μm × 0.5 cm, 5 μm, 100 Å) and desalted with solvent A for 3 min at a flow rate of 10 μL/min. After trapping, eluted peptides were separated on an EASY‐Spray analytical column (15 cm × 75 μm ID PepMap RSLC C18, 3 μm 100 Angstrom and 50 cm × 75 μm ID PepMap RSLC C18, 3 μm, 100 Å for samples from in‐solution and in situ digestions, respectively), heated to 35°C, at a flow rate of 300 nL/min by using the following gradient: 4% B for 3 min, from 4% to 22% B in 50 min, from 22% to 35% B in 10 min, from 35% to 90% B in 5 min. A washing (90% B for 5 min) and a re‐equilibration (4% B for 15 min) step was always included at the end of the gradient. Eluting peptides were analyzed on the Q‐Exactive mass spectrometer operating in positive polarity mode with capillary temperature of 280°C and a potential of 1.9 kV applied to the capillary probe. Full MS survey scan resolution was set to 70,000 with an automatic gain control (AGC) target value of 3 × 10^6^ for a scan range of 375–1500 m/z and maximum ion injection time (IT) of 100 ms. The mass at m/z 445.12003 was used as the lock mass. A data‐dependent top 5 method was operated during which higher‐energy collisional dissociation (HCD) spectra were obtained at 17500 MS2 resolution with AGC target of 1 × 10^5^ for a scan range of 200–2000 m/z, maximum IT of 55 ms, 2 m/z isolation width and normalized collisional energy (NCE) of 27. Precursor ions targeted for HCD were dynamically excluded for 15 s. Full scans and Orbitrap MS/MS scans were acquired in profile mode, whereas ion trap mass spectra were acquired in centroid mode. Charge state recognition was enabled by excluding unassigned and singly charged states. Acquired raw files were analyzed by the Thermo Scientific Proteome Discoverer 2.1 and the Thermo Xcalibur 3.1 (Thermo Fisher Scientific) software. Cross‐linked peptides were identified by manual searches of accurate multicharged peaks considering the optional oxidation of methionine. Searches were also performed with the support of the StavroX software (v. 3.6.0.1) (Götze et al. 2012) with the following parameter settings: protease sites: K, R; missed cleavage sites: K = 3, R = 2; fixed modification: carbamidomethylation (B); variable modifications: max 1 methionine oxidation (m); cross‐linker: SDA (C8O1H6); cross‐linked site 1: K, S, T, Y, N‐term; site 2: A, I, L, M, S, T, W, H, D, E, N, K, P, G, V, Q, m, C, B, Y, R, F. Precursor mass deviation: 10 ppm; fragment mass deviation: 0.02 Da; lower mass limit: 200 Da; upper mass limit: 6000 Da; S/N ratio: 2.0; ion types: b‐and y‐ions; no neutral loss. Extracted ion chromatograms of signal ions corresponding to the cross‐linked peptides were manually generated by the Thermo Xcalibur 4.6 software.

### 4.10 Cell culture and viability assays

LNCaP, DU145, CESS cell lines were purchased from American Type Culture Collection (ATCC). Cells were cultured in a humidified incubator in 5% CO_2_ at 37°C in RPMI‐1640 medium (ATCC) supplemented with 10% fetal bovine serum (FBS), (certified, One Shot™ format, United States), antibiotics (150 U/mL penicillin, 200 U/mL streptomycin) and 2 mM Glutamine (Gibco). All cell lines were routinely tested using Mycoplasma Detection Kit (Takara). Cell line authentication (STR) was carried out. The CellTiter‐Glo cell viability assay was performed according to the manufacturer's instructions (Promega). Briefly, cells were plated in opaque 96‐well plates and treated with 500 μM RYR or VRW for 96 h. After 96 h, the cells were then incubated for 10 min with CellTiter‐Glo reagent, and luminescence was measured using Packard Lumicount Microplate Reader BL10000 at 96 h.

#### 4.10.1 RNA extraction and quantitative real time PCR

Total RNA was extracted using the Direct‐zol RNA MicroPrep Kit (Zymo Research), and 1 μg of RNA was reverse transcribed using the High‐Capacity cDNA Reverse Transcription kit (Applied Biosystems). Quantitative real time PCT (qRT‐PCR) was carried out on the ABI 7500 Fast real‐time PCR machine (Life Technologies) using the TaqMan® Universal PCR Master Mix (Applied Biosystems). Experimental Ct values were normalized to β‐actin for cell lines, and the relative mRNA expression was then calculated using HEK293T as a reference sample. The following predesigned TaqMan® Gene Expression Assays were used: β‐actin Human Hs01060665_g1 FAM, Gadd45β Human Hs04188837_g1 FAM.

#### 4.10.2 Peptides uptake analysis and immunofluorescence assay

BD FACS Melody™ Cell Sorter was used to investigate the delivery of FITC‐βa‐RYR‐ and FITC‐βa‐VRW in CESS cell line. After treatment, cells were rinsed twice with PBS, centrifuged at 4°C at 3000 rpm, resuspended in 500 μL of PBS and analyzed. FACS analysis was performed using FlowJoTM (Tree Star) Software.

For immunofluorescence staining, cells treated with FITC‐βa‐RYR and FITC‐βa‐VRW at indicated time points were mounted onto poly‐L‐lysine coated slides and fixed with ice‐cold methanol‐acetone (1:1). Slides were then washed with 1× PBS and mounted using VECTASHIELD Antifade Mounting Medium with DAPI (Vector Laboratories). Images were acquired using a Leica TCS SP5 Confocal Microscopy. A 40× objective was used. Data were analyzed using GraphPad Prism version 9.0. CellTiter Glo viability assay results were expressed as means ± SD. Statistical significance was determined using a two‐tailed Student's *t* test. *p*‐values of <0.05 were considered statistically significant.

## AUTHOR CONTRIBUTIONS

**Samuele Di Cristofano:** Conceptualization; software; data curation; writing – original draft. **Emanuela Iaccarino:** Methodology; data curation. **Andrea Caporale:** Methodology; data curation. **Daniela Verzella:** Methodology; data curation. **Lucia Falcigno:** Writing – original draft; methodology; data curation. **Gabriella D'Auria:** Writing – original draft; methodology; data curation. **Rosita Russo:** Methodology; writing – original draft. **Camilla Rega:** Methodology. **Angela Chambery:** Methodology; writing – original draft. **Angela Oliver:** Methodology; data curation. **Giovannina Barisciano:** Methodology. **Simon Cross:** Methodology. **Gabriele Cruciani:** Conceptualization; methodology. **Daria Capece:** Methodology; data curation; writing – original draft. **Francesca Zazzeroni:** Methodology; resources; formal analysis. **Menotti Ruvo:** Methodology; conceptualization; data curation; writing – review and editing; writing – original draft; resources; funding acquisition; formal analysis. **Annamaria Sandomenico:** Writing – original draft; writing – review and editing; methodology; data curation; conceptualization; supervision; resources; funding acquisition; formal analysis. **Domenico Raimondo:** Data curation; supervision; software; methodology; conceptualization; funding acquisition; writing – original draft; writing – review and editing; resources.

## CONFLICT OF INTEREST STATEMENT

The authors declare no conflicts of interest.

## Supporting information


**Figure S1.** (a) Human Gadd45β structure as predicted by AlphaFold2. Each residue is colored by its predicted local distance difference test (pLDDT) value. AlphaFold2 is confident in the local structure if the pLDDT is >70. (b) Gadd45β structure with aL2 refined through Rosetta NGK protocol and without he N‐terminal tail (see section 4). (c) RMSD as a function of simulation time computed for the secondary structure Cα atoms of unbound Gadd45β (upper) and for acidic loops Cα atoms (lower). (d) Structural representation of Gadd45β cluster representatives after acidic loop clustering (see section 4). (e) Plot of RMSF values computed for each residue of unbound Gadd45β.
**Figure S2.** Comparison of Gadd45β structural models generated by different approaches and quantitative assessment of structural similarity. (a) Side and top views of Gadd45β models derived from this work (refined AF2), MODELER (template PDB: 2KG4), AlphaFold3, and Boltz‐2.2 show a conserved core fold but variation in aL1, aL2, and terminal loop regions depending on the modeling method. (b) Superposition of the four models in two orientations confirms structural agreement in the central β‐sheet and α‐helical core, with the largest deviations localized to flexible loops. MODELER produces the most compact structure, while AlphaFold3 and Boltz‐2.2 predict more extended loops. (c) A pairwise Cα RMSD heatmap reveals low RMSD (<0.2 Å) values across models, indicating overall high similarity. Cα positions of the residues used for alignment and RMSD calculation are shown on the reference structure, highlighting the conserved core region used for quantitative evaluation. Together, these analyses validate the reliability of the model generated in this work and delineate method‐dependent structural variability.
**Figure S3.** Sequence conservation and structural‐electrostatic comparison of the Gadd45 protein family. (a) Multiple sequence alignment (MSA) of human Gadd45α, Gadd45β, and Gadd45γ reveals conserved core regions and divergent aL1 and aL2 loops, suggesting protein‐specific interaction properties. (b) Structural representation of Gadd45β shows aL1 and aL2 loop placement and a pronounced negatively charged surface patch, as visualized by electrostatic potential mapping. (c) Gadd45α (PDB: 2KG4) displays a similar fold but a more neutral electrostatic surface near the loops, indicating potential differences in binding specificity. (d) Gadd45γ (PDB: 3FFM) retains an overall acidic surface similar to Gadd45β but exhibits distinct loop orientations. These structural and electrostatic variations among paralogs highlight the functional diversification of the Gadd45 family.
**Figure S4.** (a) SASA values of aromatic side chains (Tyr in RYR and Trp in VRW) sampled during the ensemble docking protocol. Dashed horizontal line represent the 20 Å^2^ cut‐off used the exclude binding modes in the “*SASA filtering step*.” (b) Molecular docking poses of RYR (upper) and VRW peptides (lower) within the P1 pocket, obtained through ensemble docking and selected according to 3 scoring functions (see section 4). (c) Group Epitope Mapping (GEM, %) analysis of D‐tripeptides RYR and (d) VRW in complex with Gadd45β at R100. The saturation of individual protons was normalized to the highest saturated proton for each peptide.
**Figure S5.** Ligand RMSD (a) and MM/GBSA energy (b) as a function of simulation time computed for RYR (upper panel) and VRW (lower panel). Replicas are colored from light to dark colors. (c) Superposition of representative RYR poses adopted multiple independent MD simulations. The pose interconversion starting from different initial binding modes is showed. Arg3 in pose #1 (r0) adopt a slightly different side chain rotamer compared to other poses.
**Figure S6.** (a–e) Extended computational flowchart showing the protocol used considering both tripeptides and Gadd45β loops flexibility during ensemble docking campaign followed by rescoring, analysis and orthogonal validation (high‐temperature MD) of binding modes using multiple independent atomistic MD simulations.
**Figure S7.** Frequency of (a) RYR and (b) VRW contacts as determined during MD simulations. Contacts less frequent than 25% of the analyzed frames were excluded from the analysis. The frame selection strategy used for this analysis is described in section 4.8.
**Figure S8.** Root‐mean‐square fluctuation (RMSF) analysis of the apo GADD45β protein and in complex with RYR and VRW peptides. Top panels show RMSF profiles of apo (gray) compared with RYR (teal) and VRW (purple), with shaded areas representing the standard error of the mean (SEM) across three replicates. Bottom panels depict the difference in RMSF (ΔRMSF) between apo and RYR or VRW bound systems, with SEM indicated as transparent bands. Transparent yellow highlights indicate the acidic loops aL1 (residues 62–68) and aL2 (residues 103–117).
**Figure S9.** Barplots show the interface RMSD values calculated according to CAPRI criteria for three representative binding poses of each peptide. Left (a) and right (b) panels correspond to RYR and VRW peptides, respectively, highlighting differences in pose stability and interface geometry. Error bars represent the standard deviation across independent simulations, providing a quantitative assessment of the variability and reliability of each binding mode.
**Figure S10.** Changes in secondary structure during standard and high temperature MD. The bar plots show the secondary structure content according to the DSSP dictionary using a concatenated trajectory with all the MD replicates.
**Figure S11.** Mutational analysis of Gadd45β aL1 and aL2 loops and their impact on RYR binding stability. (a) Side and top views of the wild‐type Gadd45β structure highlighting the aL1 and aL2 loops. Negatively charged residues (D62, D64, D65, D68, D116, E113) and the positively charged residue R115 are shown as spheres. (b) In the aL1 mutant, substitution of acidic residues impair RYR binding. The center‐of‐mass (CoM) distance between Gadd45β and RYR along MD at *T* = 310 K shows fast unbinding. (c) The aL2 mutant displays an initial stable phase followed by complete dissociation after ~100 ns. (d) The aL1–2 double mutant shows fast and complete loss of interaction, as evidenced by a rapid and persistent increase in CoM distance. Together, these results demonstrate that both aL1 and aL2 contribute cooperatively to stable peptide binding.
**Figure S12.** qRT‐PCR showing the *GADD45B* mRNA levels in CESS, DU145, and LNCaP cancer cell lines. Value denote mean ± SD.
**Figure S13.** STD spectra of G1 series/Gadd45β at increasing R molar ratios. (a) G1.1; (b) G1.2; (c) G1.4; (d) G1.5.
**Table S1.** Details about all simulated systems.

## Data Availability

The data that support the findings of this study are available from the corresponding author upon reasonable request.
