# Supplementary material for: Selection of short Gadd45β‐binding peptides through a synergistic computational and biophysical approach
Source: Protein Sci. 2025 Nov 18;34(12):e70380. doi: 10.1002/pro.70380 (PMC12624759; doi:10.1002/pro.70380)
Supplement: Supplementary file 1 — Figure S1. (a) Human Gadd45β structure as predicted by AlphaFold2. Each residue is colored by its predicted local distance difference test (pLDDT) value. AlphaFold2 is confident in the local structure if the pLDDT is >70. (b) Gadd45β structure with aL2 refined through Rosetta NGK protocol and without he N‐terminal tail (see section 4). (c) RMSD as a function of simulation time computed for the secondary structure Cα atoms of unbound Gadd45β (upper) and for acidic loops Cα atoms (lower). (d) Structural representation of Gadd45β cluster representatives after acidic loop clustering (see section 4). (e) Plot of RMSF values computed for each residue of unbound Gadd45β. Figure S2. Comparison of Gadd45β structural models generated by different approaches and quantitative assessment of structural similarity. (a) Side and top views of Gadd45β models derived from this work (refined AF2), MODELER (template PDB: 2KG4), AlphaFold3, and Boltz‐2.2 show a conserved core fold but variation in aL1, aL2, and terminal loop regions depending on the modeling method. (b) Superposition of the four models in two orientations confirms structural agreement in the central β‐sheet and α‐helical core, with the largest deviations localized to flexible loops. MODELER produces the most compact structure, while AlphaFold3 and Boltz‐2.2 predict more extended loops. (c) A pairwise Cα RMSD heatmap reveals low RMSD (<0.2 Å) values across models, indicating overall high similarity. Cα positions of the residues used for alignment and RMSD calculation are shown on the reference structure, highlighting the conserved core region used for quantitative evaluation. Together, these analyses validate the reliability of the model generated in this work and delineate method‐dependent structural variability. Figure S3. Sequence conservation and structural‐electrostatic comparison of the Gadd45 protein family. (a) Multiple sequence alignment (MSA) of human Gadd45α, Gadd45β, and Gadd45γ reveals conserved core regions [file PRO-34-e70380-s001.docx]

**Selection of short GADD45β-binding peptides through a synergistic computational and biophysical approach.**

Samuele Di Cristofano^1,7^, Emanuela Iaccarino^2,7^, Andrea Caporale^3^, Daniela Verzella^4^, Lucia Falcigno^5^, Gabriella D’Auria^5^, Rosita Russo^6^, Camilla Rega^6^, Angela Chambery^6^, Angela Oliver^1^, Giovannina Barisciano^1^, Daria Capece^4^ Francesca Zazzeroni^4^, Menotti Ruvo^2^, Annamaria Sandomenico^2,8^ and Domenico Raimondo ^1,8^

^1^ Department of Molecular Medicine, Sapienza University of Rome, Laboratory affiliated to Istituto Pasteur Italia – Fondazione Cenci Bolognetti, Rome, Italy - Viale Regina Elena 291, 00161 - Rome,
^2^ Institute of Biostructures and Bioimaging (IBB), National Research Council (CNR), Via P. Castellino 111, 80131 Naples, Italy
^3^ Institute of Crystallography (IC), National Research Council (CNR), Strada Statale 14 km 163.5, Basovizza, 34149 Trieste, Italy
^4^ Department of Biotechnological and Applied Clinical Sciences, University of L'Aquila, 67100 L'Aquila, Italy
^5^ Department of Pharmacy, University Federico II of Naples, 80131 Naples, Italy.
^6^ Department of Environmental, Biological and Pharmaceutical Science and Technology, University of Campania "Luigi Vanvitelli", 81100, Caserta, Italy.

**– Supplementary material –**


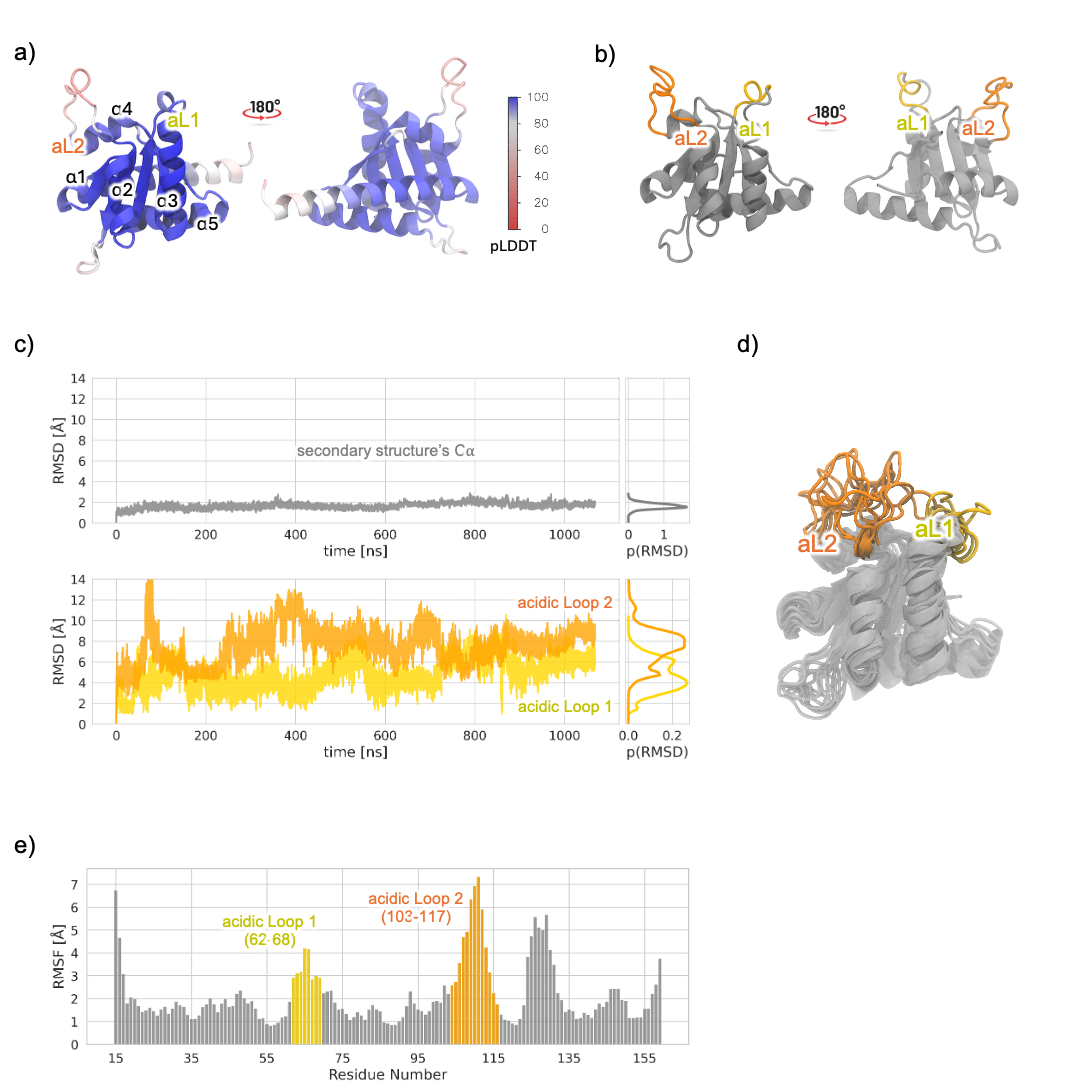


**Supplementary Fig. S1 a)** Human Gadd45β structure as predicted by AlphaFold2. Each residue is colored by its predicted local distance difference test (pLDDT) value. AlphaFold2 is confident in the local structure if the pLDDT is >70. **b)** Gadd45β structure with aL2 refined through Rosetta NGK protocol and without he N-terminal tail (see Methods). **c)** RMSD as a function of simulation time computed for the secondary structure Cα atoms of unbound Gadd45β (upper) and for acidic loops Cα atoms (lower). **d)** Structural representation of Gadd45β cluster representatives after acidic loop clustering (see Methods). **e)** Plot of RMSF values computed for each residue of unbound Gadd45β


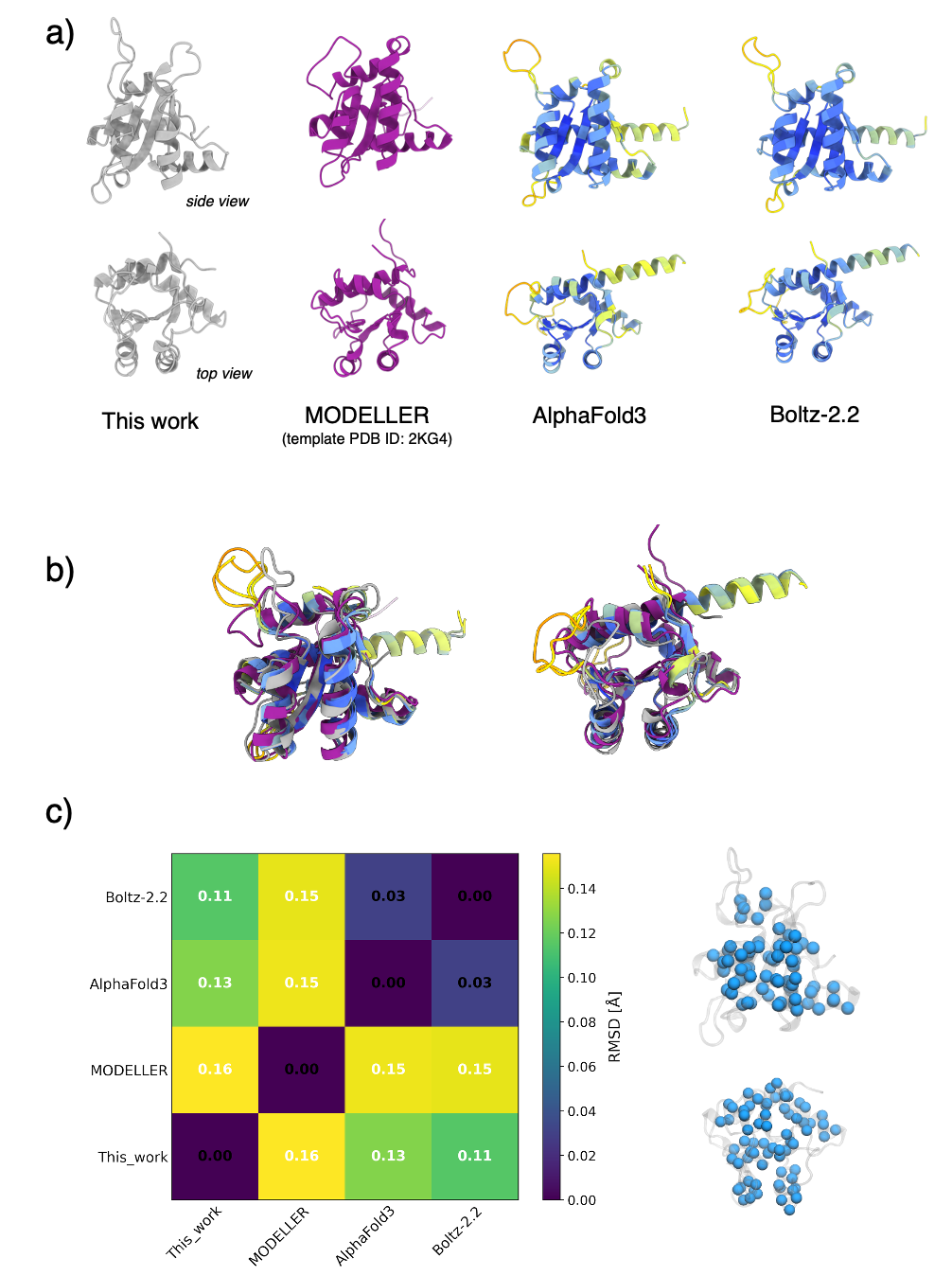


**Supplementary Fig. S2**. Comparison of Gadd45β structural models generated by different approaches and quantitative assessment of structural similarity. **a)** Side and top views of Gadd45β models derived from this work (refined AF2), MODELLER (template PDB: 2KG4), AlphaFold3 and Boltz-2.2 show a conserved core fold but variation in aL1, aL2 and terminal loop regions depending on the modeling method. **b)** Superposition of the four models in two orientations confirms structural agreement in the central β-sheet and α-helical core, with the largest deviations localized to flexible loops. MODELLER produces the most compact structure, while AlphaFold3 and Boltz-2.2 predict more extended loops. **c)** A pairwise Cα RMSD heatmap reveals low RMSD (< 0.2 Å) values across models, indicating overall high similarity. Cα positions of the residues used for alignment and RMSD calculation are shown on the reference structure, highlighting the conserved core region used for quantitative evaluation. Together, these analyses validate the reliability of the model generated in this work and delineate method-dependent structural variability.


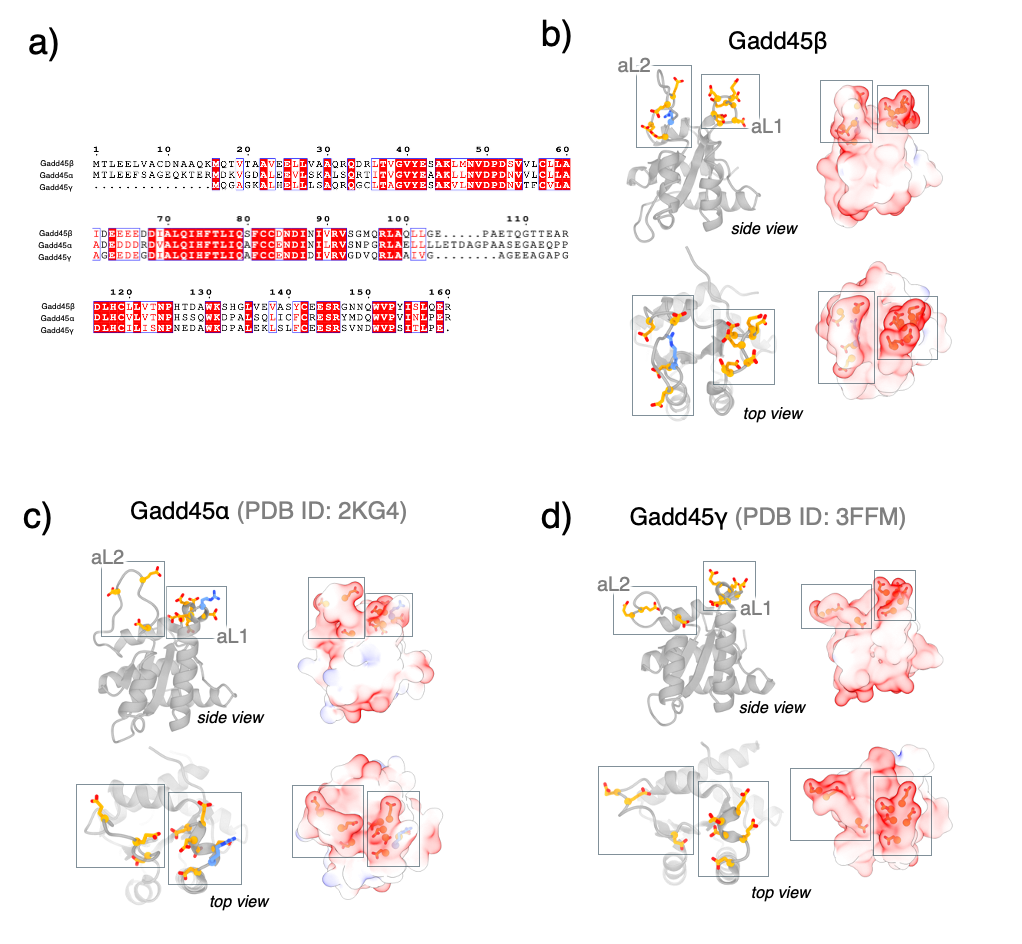


**Supplementary Fig. S3**. Sequence conservation and structural-electrostatic comparison of the Gadd45 protein family. **a)** Multiple sequence alignment (MSA) of human Gadd45α, Gadd45β and Gadd45γ reveals conserved core regions and divergent aL1 and aL2 loops, suggesting protein-specific interaction properties. **b)** Structural representation of Gadd45β shows aL1 and aL2 loop placement and a pronounced negatively charged surface patch, as visualized by electrostatic potential mapping. **c)** Gadd45α (PDB: 2KG4) displays a similar fold but a more neutral electrostatic surface near the loops, indicating potential differences in binding specificity. **d)** Gadd45γ (PDB: 3FFM) retains an overall acidic surface similar to Gadd45β but exhibits distinct loop orientations. These structural and electrostatic variations among paralogs highlight the functional diversification of the Gadd45 family.


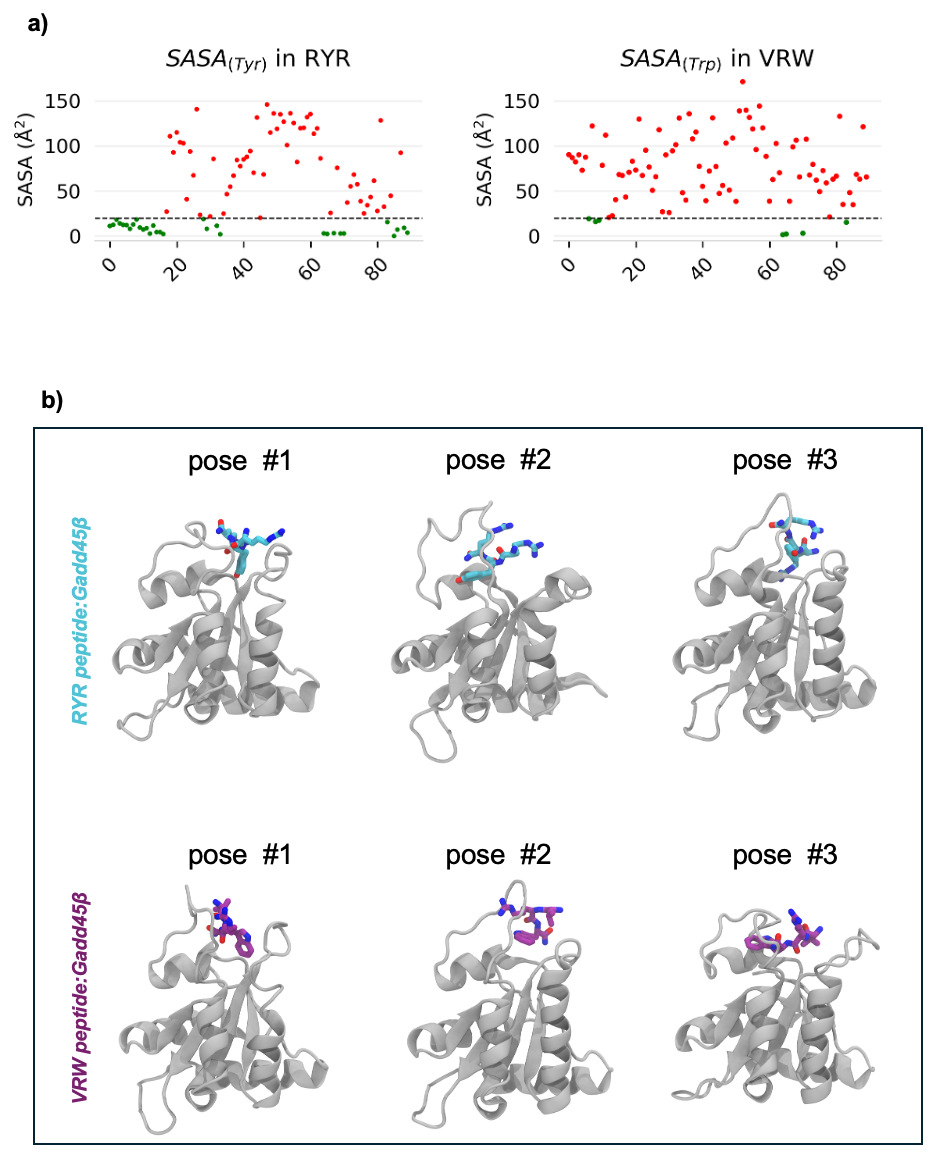


**c)**


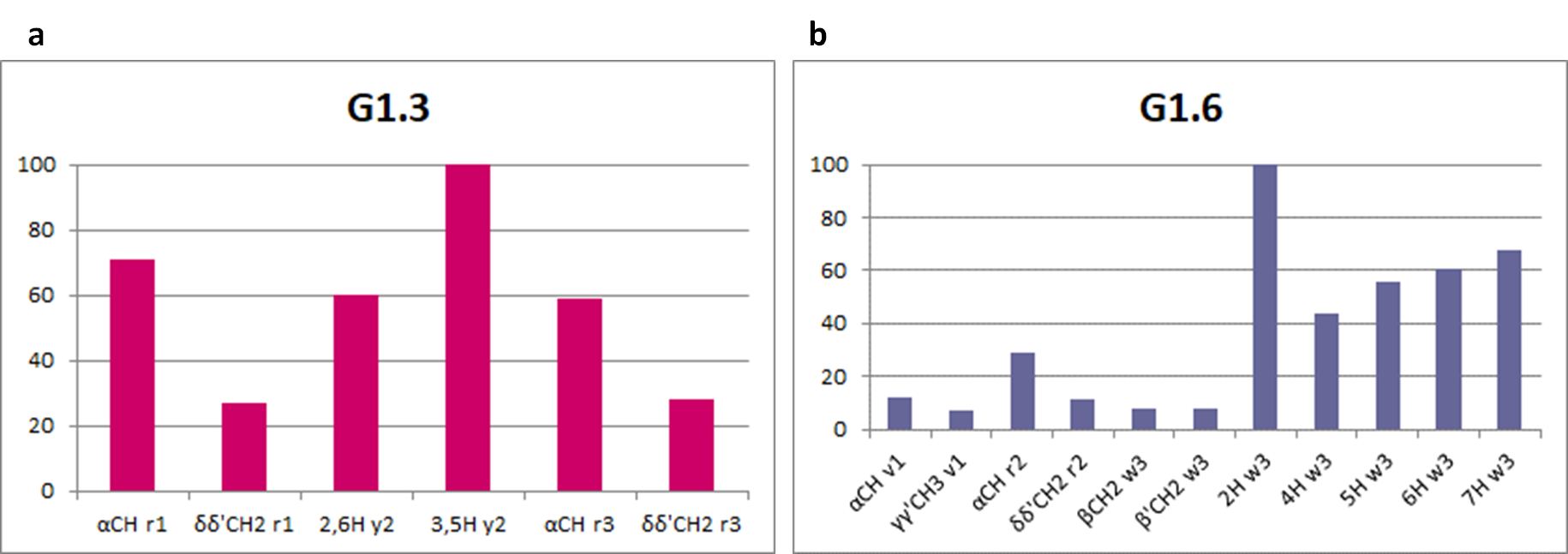


**Supplementary Fig. S4**  **a)** SASA values of aromatic side chains (Tyr in RYR and Trp in VRW) sampled during the ensemble docking protocol. Dashed horizontal line represent the 20 Å^2^ cut-off used the exclude binding modes in the “*SASA filtering step”.* **b)** Molecular docking poses of RYR (upper) and VRW peptides (lower) within the P1 pocket, obtained through ensemble docking and selected according to 3 scoring functions (see Methods). **c)** Group Epitope Mapping (GEM, %) analysis of D-tripeptides RYR and **d**) VRW in complex with Gadd45β at R100. The saturation of individual protons was normalized to the highest saturated proton for each peptide.


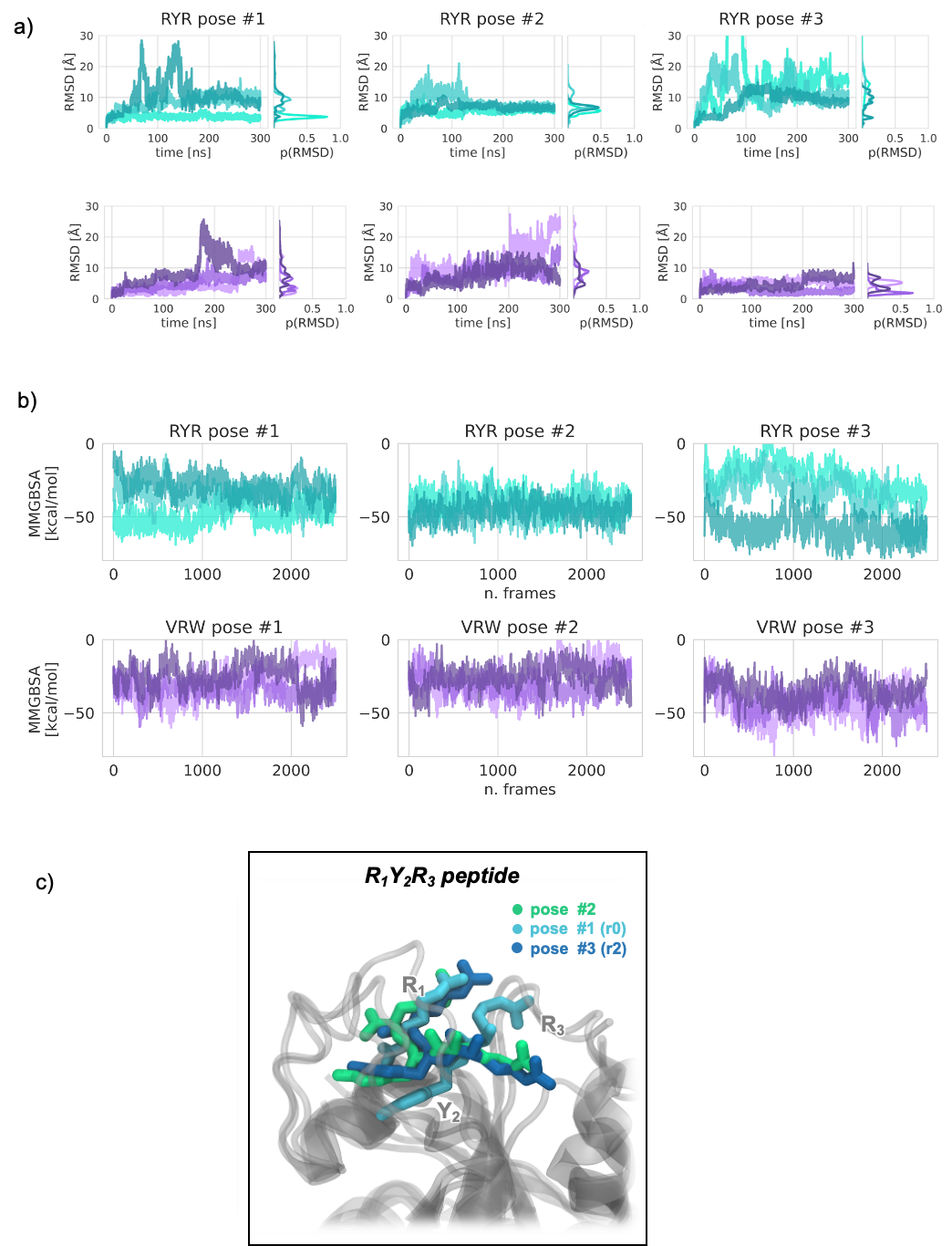


**Supplementary Fig. S5** Ligand RMSD. a) and MM/GBSA energy b) as a function of simulation time computed for RYR (upper panel) and VRW (lower panel). Replicas are colored from light to dark colors. c) Superposition of representative RYR posesSuperposition of representative RYR poses adopted multiple independent MD simulations. The pose interconversion starting from different initial binding modes is showed. Arg3 in pose #1 (r0) adopt a slightly different side chain rotamer compared to other poses.


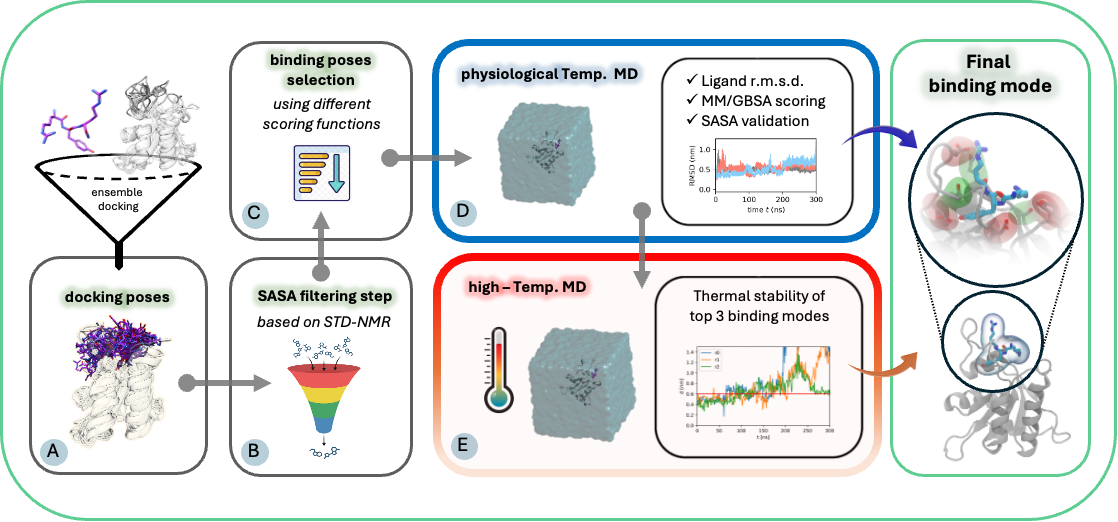


**Supplementary Fig. S6 a-e** Extended computational flowchart showing the protocol used considering both tripeptides and Gadd45β loops flexibility during ensemble docking campaign followed by rescoring, analysis and orthogonal validation (high-temperature MD) of binding modes using multiple independent atomistic MD simulations.


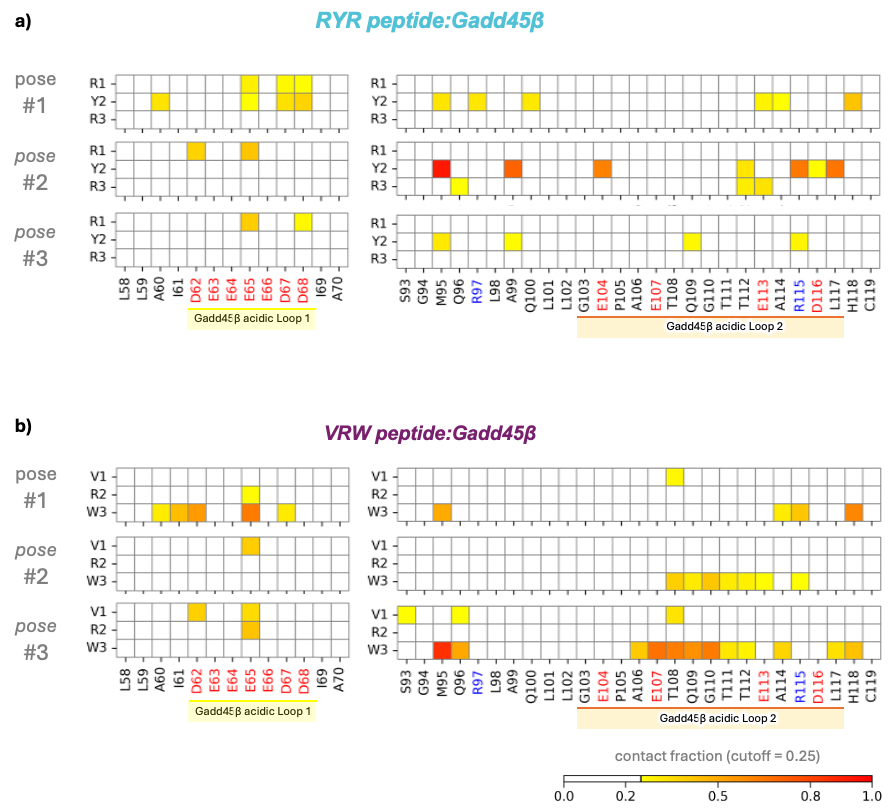


**Supplementary Fig. S7.**  Frequency of **a)** RYR and **b**) VRW contacts as determined during MD simulations. Contacts less frequent than 25 % of the analyzed frames were excluded from the analysis. The frame selection strategy used for this analysis is described in the “*Molecular Mechanics / Generalized Born Surface Srea (MM/GBSA)*” section of Methods paragraph.


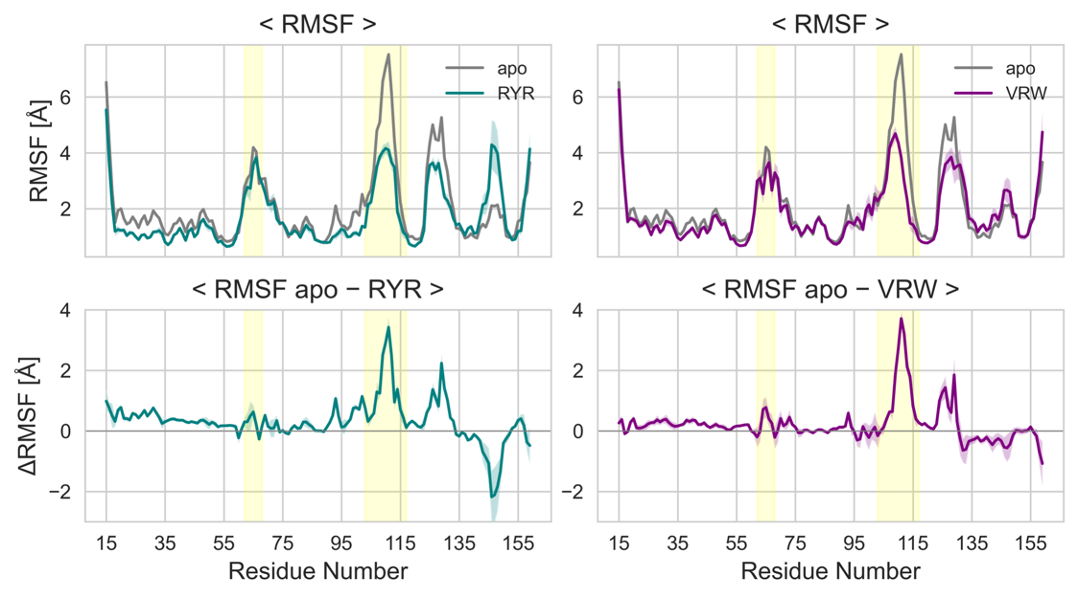


**Supplementary Fig. S8**. Root-mean-square fluctuation (RMSF) analysis of the apo GADD45β protein and in complex with RYR and VRW peptides. Top panels show RMSF profiles of apo (gray) compared with RYR (teal) and VRW (purple), with shaded areas representing the standard error of the mean (S.E.M.) across 3 replicates. Bottom panels depict the difference in RMSF (ΔRMSF) between apo and RYR or VRW bound systems, with S.E.M. indicated as transparent bands. Transparent yellow highlights indicate the acidic loops aL1 (residues 62–68) and aL2 (residues 103–117).


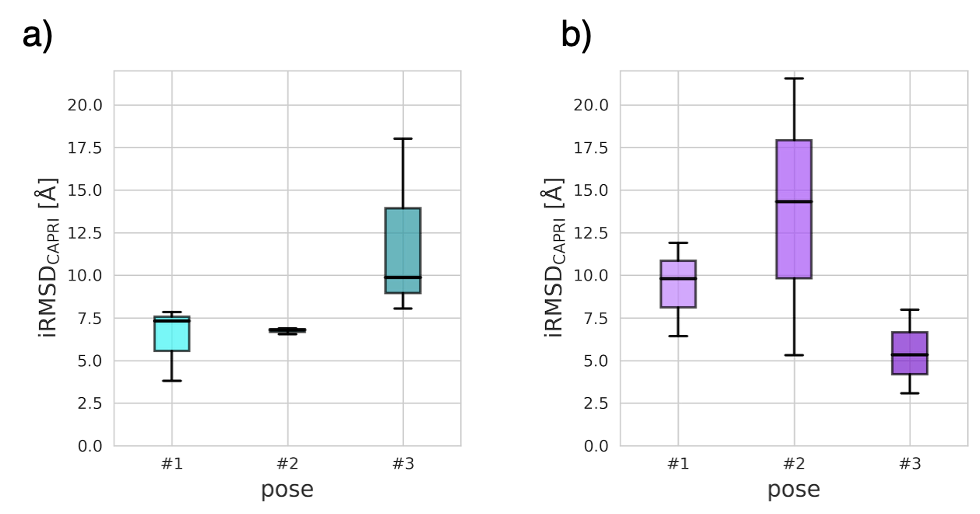


**Supplementary Fig. S9**. Barplots show the interface RMSD values calculated according to CAPRI criteria for 3 representative binding poses of each peptide. Left **a)** and right **b)** panels correspond to RYR and VRW peptides, resectively,highlighting differences in pose stability and interface geometry. Error bars represent the standard deviation across independent simulations, providing a quantitative assessment of the variability and reliability of each binding mode.

**a)**


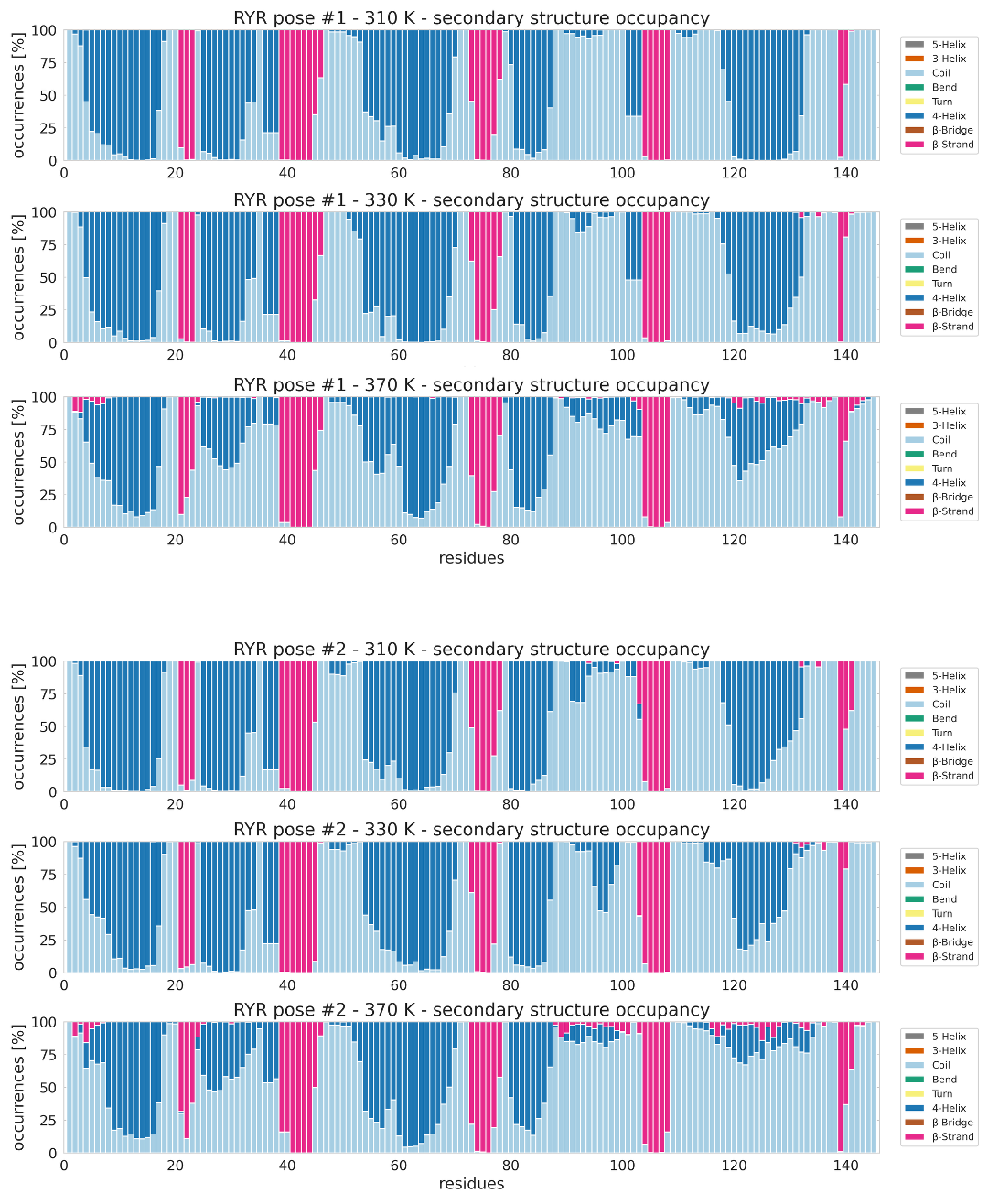


**b)**


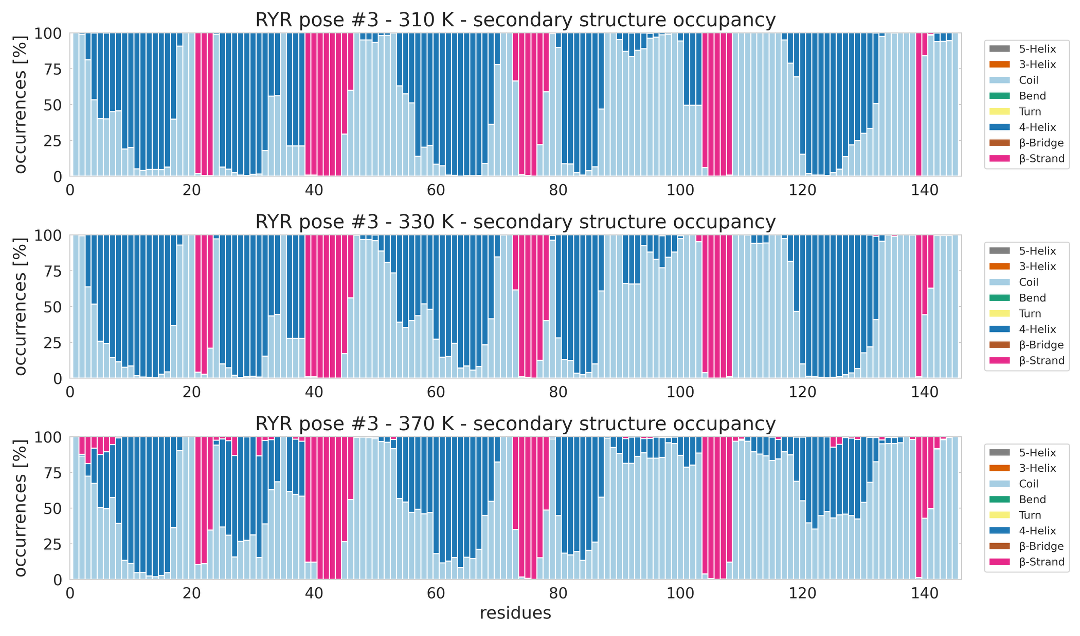


**Supplementary Fig. S10.** Changes in secondary structure during standard and high temperature MD. The bar plots show the secondary structure content according to the DSSP dictionary using a concatenated trajectory with all the MD replicates.


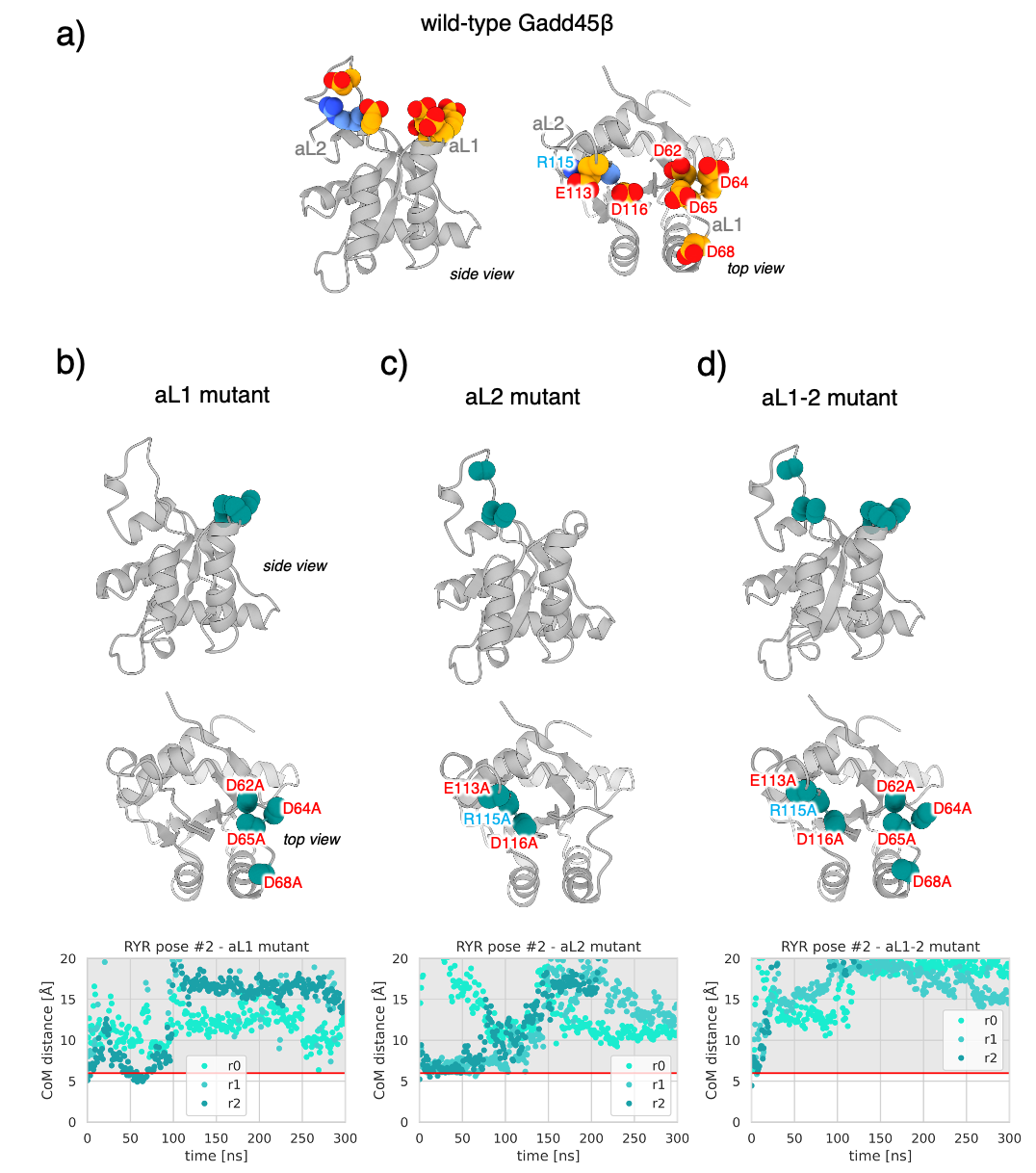


**Supplementary Fig. S11.** Mutational analysis of Gadd45β aL1 and aL2 loops and their impact on RYR binding stability. **a)** Side and top views of the wild-type Gadd45β structure highlighting the aL1 and aL2 loops. Negatively charged residues (D62, D64, D65, D68, D116, E113) and the positively charged residue R115 are shown as spheres. **b)** In the aL1 mutant, substitution of acidic residues impair RYR binding. The center-of-mass (CoM) distance between Gadd45β and RYR along MD at T=310K shows fast unbinding. **c)** The aL2 mutant displays an initial stable phase followed by complete dissociation after ~100 ns. **d)** The aL1–2 double mutant shows fast and complete loss of interaction, as evidenced by a rapid and persistent increase in CoM distance. Together, these results demonstrate that both aL1 and aL2 contribute cooperatively to stable peptide binding.


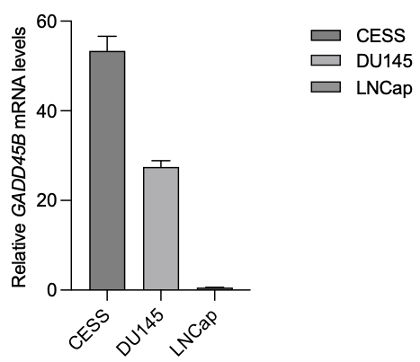


**Supplementary Fig. S12**. qRT-PCR showing the *GADD45B* mRNA levels in CESS, DU145 and LNCap cancer cell lines. Value denote mean ± SD.


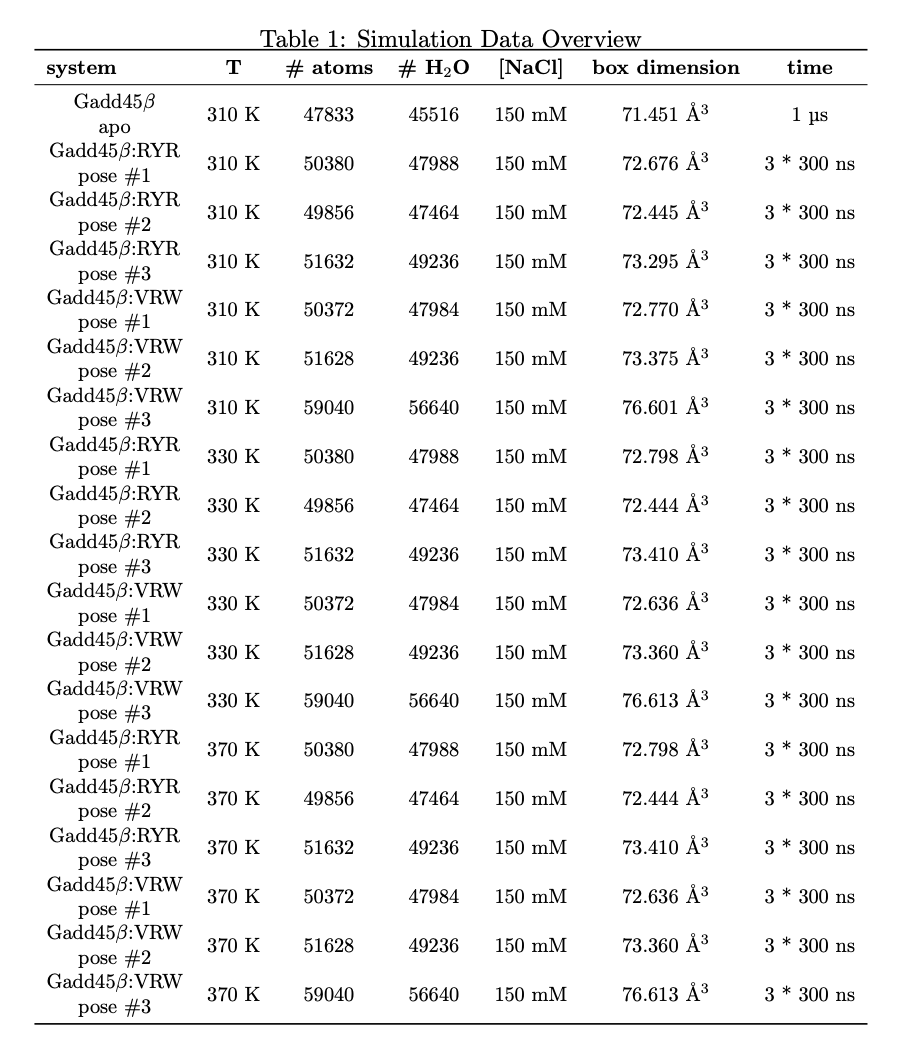


**Supplementary Table 1.** Details about all simulated systems


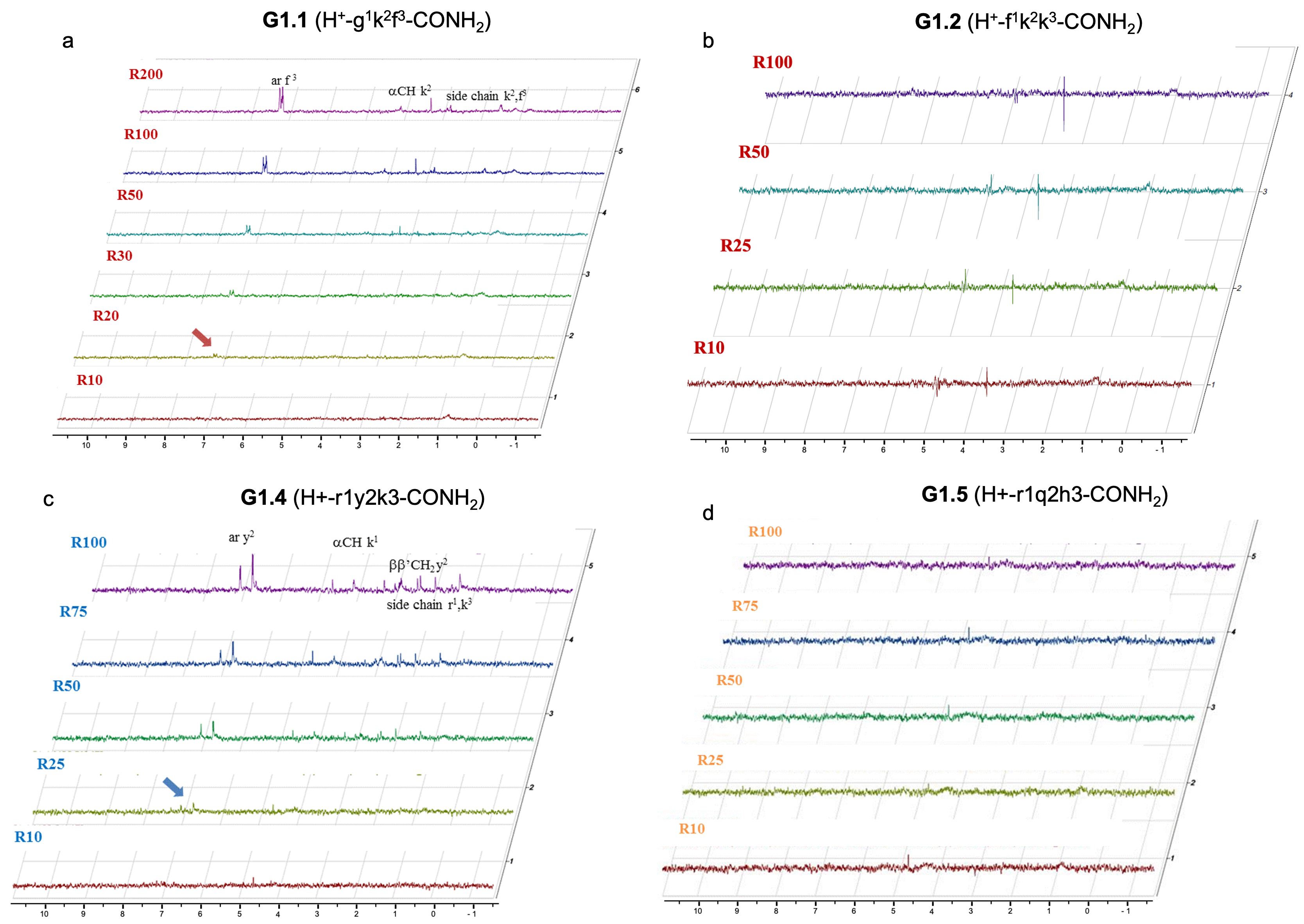


f1 (ppm)

f1 (ppm)

f1 (ppm)

f1 (ppm)

**Supplementary Fig. S13**. STD spectra of G1 series/Gadd45β at increasing R molar ratios. **a**) G1.1; **b**) G1.2; **c**) G1.4; **d**) G1.5;
